# Sur-X, a novel peptide, kills colorectal cancer cells by targeting survivin-XIAP complex

**DOI:** 10.1186/s13046-020-01581-3

**Published:** 2020-05-07

**Authors:** Wanxia Fang, Xiaofang Che, Guohui Li, Anhui Wang, Yizhe Wang, Xiaonan Shi, Kezuo Hou, Xiaojie Zhang, Xiujuan Qu, Yunpeng Liu

**Affiliations:** 1 Department of Medical Oncology, The First Hospital of China Medical University, Shenyang, 110001 China; 2 Key Laboratory of Anticancer Drugs and Biotherapy of Liaoning Province, The First Hospital of China Medical University, Shenyang, 110001 China; 3 Liaoning Province Clinical Research Center for Cancer, The First Hospital of China Medical University, Shenyang, 110001 China; 4 Key Laboratory of Precision Diagnosis and Treatment of Gastrointestinal Tumors, Ministry of Education, The First Hospital of China Medical University, Shenyang, 110001 China; 5 Laboratory of Molecular Modeling and Design, State Key Laboratory of Molecular Reaction Dynamics, Dalian Institute of Chemical Physics, Chinese Academy of Sciences, Dalian, 116024 China; 6 State Key Laboratory of Fine Chemicals, School of Chemistry, Dalian University of Technology, Dalian, 116024 China; 7 Department of Respiratory and Infectious Disease of Geriatrics, The First Hospital of China Medical University, Shenyang, 110001 China

**Keywords:** Colorectal cancer, Survivin-XIAP complex, Anticancer peptide, Apoptosis, Necroptosis

## Abstract

**Background:**

Survivin and XIAP are two important members of the inhibitor of apoptosis protein family and have been considered as potential targets for cancer treatment due to their overexpression in large variety of cancers including colorectal cancer. It has been reported that survivin and XIAP can synergistically inhibit apoptosis by forming survivin-XIAP complex. In this study, we aimed to design a peptide that targets the survivin-XIAP complex and elucidate its anticancer mechanisms in colorectal cancer cells.

**Methods:**

We designed and synthetized Sur-X, the peptide targeting survivin-XIAP complex. The anticancer effects of Sur-X were evaluated both in vitro and in vivo. The underlying molecular mechanisms were also investigated.

**Results:**

Sur-X exhibited potent inhibitory effects on four colorectal cancer cell lines HCT116, HCT15, RKO and HT29, but not on human peritoneal mesothelial cell line HMrSV5. Mechanistically, Sur-X induced Caspase 9-dependent intrinsic apoptosis in colorectal cancer cells by disrupting the survivin-XIAP complex and subsequently destabilizing survivin and XIAP. Interestingly, we found that Sur-X can also promote necroptosis. It was demonstrated that Sur-X destroyed the interaction between XIAP and TAB1 in the XIAP-TAB1-TAK1 complex, leading to the instability of TAK1, an endogenous necroptosis inhibitor. Subsequently, the accelerated degradation of TAK1 attenuated its inhibition on necroptosis in colorectal cancer cells. Moreover, knockdown of TAK1 restored the sensitivity of TAB1-overexpressing colorectal cancer cells to Sur-X-induced necroptosis. The in vivo pro-apoptotic effect of Sur-X was confirmed by the enhanced TUNEL staining and the decreased expression of survivin and XIAP in tumor tissues from xenograft mouse models. In addition, extensive necrosis and weaker MLKL expression in xenografts provided evidence for the in vivo pro-necroptotic effect of Sur-X.

**Conclusions:**

Peptide Sur-X exhibits strong pro-apoptotic and pro-necroptotic effects in colorectal cancer cells and has a high clinical translation potential in the treatment of colorectal cancer.

## Background

Colorectal cancer is the third most common cancer worldwide, with a mortality of 9.2% [1]. At present, fluorouracil and platinum-based chemotherapy is still the main treatment for advanced and recurrent colorectal cancer [2]. Although target drugs and immune checkpoint inhibitors are successful in colorectal cancer treatment, most patients still cannot benefit from these novel drugs [3]. Therefore, it is necessary to develop effective target drugs for colorectal cancer.

Survivin is the smallest member of inhibitor of apoptosis protein (IAP) family. It is overexpressed in most cancers including colorectal cancer, but rarely expressed in normal differentiated tissues [4]. Survivin contains a single N-terminal baculovirus IAP repeat (BIR) domain, a zinc finger fold and a C-terminal α helical coiled-coil domain, and exists in both monomeric and dimeric forms. Through its interactions with multiple essential proteins such as Hsp90, SMAC/DIABLO, XIAP, and the components of chromosomal passenger complex (CPC), survivin exerts two major functions: (i) inhibiting apoptosis and (ii) regulating cell division, both of which play key roles in the development of cancer [5–7]. Therefore, survivin is believed to have clinical significance and has been extensively studied. In glioma, a positive association was observed between survivin expression and pathological grade [8]. In patients with non-small-cell lung cancer, the overexpression of survivin was significantly related to poor postoperative survival [9]. It has also been reported that survivin contributes to the resistance of cancer cells to radiotherapy and chemotherapy [10, 11]. Moreover, high survivin expression in circulating tumor cells (CTC) predicted shortened overall survival in metastatic colorectal cancer patients [12]. Taken together, due to its specific overexpression in most cancer tissues, dual role in cell division regulation and apoptosis inhibition, and clinical significance, survivin has been established as an ideal target for anticancer therapy.

To date, a variety of drugs targeting survivin have been developed including YM155, LY2181308, LQZ-7F, shepherdin, survivin-specific SMAC mimetics and so on [13–17]. Inhibiting the expression of survivin or blocking the interactions between survivin and other essential proteins to interfere with its functions are two major approaches by which survivin inhibitors exert their anticancer activity [18]. Despite exhibiting potent anticancer effects both in vitro and in vivo, existing survivin-targeted drugs have not been successfully translated into the clinic [19–22]. Thus, it is necessary to develop new drugs that target survivin in cancer cells. XIAP is another important member of the IAP family. It has been reported that survivin and XIAP can form an IAP-IAP complex to synergistically antagonize the activity of Caspase 9 [23]. Therefore, we hypothesized that interfering with the interaction between survivin and XIAP may be a promising approach for colorectal cancer treatment.

In this study, we designed and synthesized a novel anticancer peptide Sur-X that targets the survivin-XIAP complex. Sur-X was proved to have specific and potent anticancer activity both in colorectal cancer cell lines and xenograft mouse models. Mechanistically, it was found that apart from inducing apoptosis by disrupting the survivin-XIAP complex, Sur-X also promoted necroptosis in colorectal cancer cells by interfering with the interaction between XIAP and TAB1. Thus, this study provides a potential strategy for colorectal cancer treatment.

## Materials and methods

### On-line databases analysis

The mRNA expression levels of IAPs in cancer tissues were compared with normal controls by Oncomine (https://www.oncomine.org/), an on-line cancer microarray database. The thresholds of *p*-value and fold change were set to 0.05 and 1.5, respectively.

GEPIA (Gene Expression Profiling Interactive Analysis) is an interactive web server for analyzing gene expression profiling of cancer and normal tissues from The Cancer Genome Atlas (TCGA) and the Genotype-Tissue Expression (GTEx) projects (http://gepia.cancer-pku.cn/). The expression profile of BIRC5/survivin in multiple cancers and all the IAPs expression in colorectal cancer and matched normal tissues were analyzed by GEPIA, parameters were set to default values.

### Reagents and antibodies

The following primary antibodies were used for Western blot: antibodies against survivin (#2803), XIAP (#14334), Caspase 8 (#9746S), Caspase 9 (#9508S), Caspase 3 (#9662S), cleaved-Caspase 3 (#9661S), PARP (#9542 L), ubiquitin (#3933S), RIP1 (#3493S), phospho-RIP1 (Ser166) (#44590), RIP3 (#13526S), phospho-RIP3 (Ser227) (#93654), phospho-MLKL (Ser358) (#91689S), TAB1 (#3226), TAK1 (#5206), phospho-TAK1 (Thr184/187) (#4508) and GAPDH (#5174) were from Cell Signaling Technology; anti-Caspase 7 antibody (#sc-28,295) was purchased from Santa Cruz and anti-MLKL antibody (#184718) was purchased from and Abcam. Z-VAD-fmk (Sigma-Aldrich, V116) and Nec-1 s (Biovision, 2535–1) were used to reveal apoptotic and necroptotic cell death, respectively. Cycloheximide (CHX, C7698-5G) was purchased from Sigma-Aldrich. MG132 (S2619) was obtained from Selleckchem.

### Cell culture

Human colorectal cancer cell lines HCT116, RKO, HCT15, and HT29 were obtained from the Type Culture Collection of the Chinese Academy of Sciences (Shanghai, China). The human peritoneal mesothelial cell line HMrSV5 (SV5) was provided by Prof. Huimian Xu (Department of Surgical Oncology and General Surgery, The First Hospital of China Medical University). All cell lines were cultured with RPMI-1640 containing 10% fetal bovine serum (FBS, Gibco, Gaithersburg, MD, USA) and 1% penicillin/streptomycin. The short tandem repeats (STR) profile was used to authenticate all the cell lines. All cells were cultured for no longer than 2 months and negatively tested for mycoplasma contamination.

### Peptide synthesis

The peptide with the sequence derived from the XIAP-binding region (K15-M38) of survivin and the cell-penetrating sequence from HIV Tat protein added to its N-terminal, was named Sur-X. The sequence of Sur-X is YGRKKRRQRRRKDHRISTFKNWPFLEGCACTPERM-COOH. A negative control peptide is YGRKKRRQRRRKDDGNYKTRAEVKFEGDTLVNRIE-COOH, and named Con. Peptides Sur-X and Con, as well as FITC labeled peptides FITC-Sur-X and FITC-Con were all synthesized by Synpeptide Co. Ltd. (Nanjing, China). DMSO was used to dissolve these peptides immediately before use. Final concentrations of DMSO in experiments were < 0.4%.

### Confocal imaging

Cells were seeded into 2 cm glass-bottom cell culture dish (NEST, 801001) and treated by 10 μM FITC-Sur-X or FITC-Con after attachment. Hoechst 33342 (BD Biosciences, 561,908) was used to stain nuclei. After 30 min, the culture medium was discarded and cells were washed twice using ice-cold PBS and inspected under an Olympus FluoView FV1000 laser scanning confocal microscope.

### Co-immunoprecipitation and Western blot analysis

When the confluency reached 90% in a 10-cm dish, cells were lysed in 1% Triton lysis buffer (1% Triton X-100, 50 mM Tris-Cl pH 7.4, 150 mM NaCl, 10 mM EDTA, 100 mM NaF, 1 mM Na_3_VO_4_, 1% protease inhibitor cocktail) on ice for 5 min and collected in a 1.5 ml EP tube. Retaining 10% of cell lysate as input, the remaining was subjected to co-immunoprecipitation assay and Western blot analysis, which were performed as described in our previous studies [24, 25]. Image J (NIH, USA) was used to perform densitometric analysis.

### Cell viability assay

3-(4,5-dimethyl thiazol-2-yl)-2,5-diphenyl tetrazolium bromide (MTT) assay was used to detect cell viability as previously described [24].

### Colony formation

Cells were seeded into 12-well plates and treated by 10 μM Sur-X or Con. Ten days later, cells were stained by Wright-Giemsa after 75% ethanol-fixation. The colonies in each well were counted in five fields under an optical microscope.

### qRT-PCR assay

Trizol-chloroform was used to extract total RNA and qRT-PCR was performed as previously described [26]. Primers were listed in Table S1.

### Real-time apoptosis and necrosis assay

HCT116 and RKO cells were seeded at the density of 4000 cells/well in 100 μl culture medium into Nunclon 96-Well flat white plate (Thermo Fisher Scientific). After cells were attached, 2× Sur-X of finally detected concentrations and 2× detection reagents in RealTime-Glo™ Annexin V Apoptosis and Necrosis Assay (Promega, JA1011) were mixed in 100 μl pre-warming culture medium and the mixture was added to each well. Relative luminescence units (RLU) and relative fluorescence units (RFU) were recorded overtime up to 6 h by the Spark multimode microplate reader (Tecan Trading AG, Switzerland).

### Caspase 3 activity assay

The activity of Caspase 3 was measured according to the instruction of Caspase 3 Activity Assay Kit (Cell Signaling Technology, #5723). RFU with excitation at 380 nm and emission at 440 nm was detected by Thermo Scientific Varioskan Flash.

### Ubiquitination assay

HCT116 cells were incubated with 10 μM MG132 for 8 h and then treated by 10 μM Sur-X for 1 h. Subsequently, immunoprecipitation was implemented as previously described. After SDS-PAGE, the immune-precipitates were probed with indicated antibodies including anti-ubiquitin antibody. The experiment was performed for three times independently.

### Annexin V-FITC/7-AAD assay

After indicated treatment, HCT116 and RKO cells were collected and prepared for flow cytometry analysis by using FITC Annexin V Apoptosis Detection Kit with 7-AAD (Biolegend, 640,922), according to the staining procedure recommended by the manufacture. BD Accuri C6 Flow Cytometer was used for the detection and analysis of samples.

### Preparation of the structures of XIAP and Sur-X for molecular dynamics simulation

To predict the binding mode of XIAP and Sur-X, the structure of XIAP was constructed based on the crystal structures of BIR1, BIR2, BIR3 domains (PDB codes: 4OXC, 4J3Y, and 1G73) and the missing residues were filled using YASARA. The assembled XIAP structure was then subject to three step-wise rounds of energy minimization to relax the loops that connect different domains. For Sur-X, we extracted corresponding atomic coordinates (residues number 15 to 38) from the crystal structure of survivin (PDB code: 1F3H). Starting from this conformer, a total of 400 ns accelerated molecular dynamics (aMD) simulations were carried out and the center of the highest-populated cluster obtained from aMD trajectories was selected as the representative low-energy conformational state of the peptide [27, 28]. Afterwards, the initial XIAP-peptide complexes were created by using ZDOCK [29]. Finally, the binding poses were clustered into 27 complexes and selected for further molecular dynamics (MD) simulations.

### MD simulation of interaction between Sur-X and XIAP

All MD simulations were performed by using Amber 16 program (University of California, San Francisco) on GPUs. The Amber99sb force field was used for all systems [30]. For each binding pose, the complex structure was solvated in a cubic periodic box of TIP3P water with at least 10 Å distance from the complex surface, followed by the addition of 0.15 M NaCl ions to neutralize the system. To relax the whole system, the starting structure was then subject to energy minimization and two rounds of 500-ps constant volume (NVT) and 500-ps constant pressure (NPT) equilibration with position restraints on the solute heavy atoms. Subsequently, the production NPT simulations were conducted for up to 200 ns for each complex system. During the production runs, all bonds involving hydrogen were constrained using the SHAKE algorithm to enable an integration time-step of 2 fs. The non-bonded interaction was cut off at 1.0 nm, whereas the electrostatic interaction beyond that was treated using the particle mesh Ewald (PME) method. The Langevin Thermostat was applied to maintain the temperature at 300 K, and the Berendsen Barostat was used to maintain the pressure at 1.0 bar. The trajectories of the production runs were saved at 8 ps intervals.

We collected 675,000 coordinates from all of the 5.4 μs production runs and performed our analysis on the basis of these snapshots. The interaction residues between XIAP and Sur-X were determined by the following criterion: if the shortest distance between any heavy atoms from two residues belonging to two proteins was less than 4.5 Å, then these two residues from two proteins were considered to form an interaction residue pair. By counting the highest-frequent pairs appeared during the 60–100, 100–160, and 160–200 ns trajectories of each simulation, we determined the interaction pairs shared between the three time periods as the interface residues between XIAP and Sur-X.

### Enzyme-linked immunosorbent assay (ELISA)

According to the manufacturer’s instruction, Human TNF-α ELISA kit (R & D Systems, DTA00D) was used to measure the TNF-α in culture medium secreted by colorectal cancer cells.

### Plasmid and small interfering RNA transfection

TAB1 plasmid and the vector pcDNA3.1(+) were purchased from Obio Technology Corp., Ltd. (Shanghai, China). SiRNAs of TAK1 and negative control (NC) were obtained from Beijing ViewSolid Biotech, the sequences are as follows: 5′-GGUGCUGAACCAUUGCCAUTT-3′ for si#1 of TAK1; 5′-GCAACCCAAAGCGCUAAUUTT-3′ for si#2 of TAK1; 5′-UUCUCCGAACGUGUCACGUTT-3′ for NC. Cells were transfected as previously described [25].

### Xenograft mouse model

A total of 10 four-week old female BALB/c nude mice were purchased from Beijing Vital River Laboratory Animal Technology Co., Ltd. (Beijing, China) and fed at a specific pathogen-free environment in Animal Laboratory Unit of China Medical University, with the approval (2019088) of Institutional Review Board of China Medical University for all in vivo experiments. HCT116 cells (5 × 10^6^) were injected subcutaneously near the right scapula of mice. When the average tumor volume reached 50–75 mm^3^, mice were randomly divided into two groups (*n* = 5) and received intravenous injection of 50 mg/kg Sur-X or Con daily for a total of 14 times since then. Tumor size and body weight were measured every other day. The volume of each tumor *V* was calculated using *V* = 1/2 (length × width^2^). Mice were killed by cervical dislocation according to the protocol filed with the Guidance of Institutional Animal Care and Use Committee of China Medical University.

### Immunohistochemistry (IHC)

Tumor tissues were fixed by formalin, embedded by paraffin and prepared for staining with haematoxylin and eosin (HE) and antibodies of survivin, XIAP, and MLKL as described in our previous studies [31]. The staining was evaluated by scanning the entire tissue specimen under low magnification (× 10) and confirmed under high magnification (× 20 and × 40). Both staining intensity and staining area were used to classify the expression of proteins, with staining intensity scored as 0 (no), 1 (low), 2 (intermediate), and 3 (high) points and staining area scored as 0 (≤5%), 1 (5–25%), 2 (25–50%), 3 (50–75%) and 4 (> 75%) points, respectively. Histoscore was calculated as histoscore = staining intensity × staining area. Two pathologists were responsible for determining the final histoscore independently. The expression of protein with a histoscore of 0, 1–4 points and 6–12 points were defined as negative (−), weak positive (+) and strong positive (++), respectively and evaluated under 5 randomly selected, non-overlapping fields from the stained sections.

### TUNEL assay

One Step TUNEL Apoptosis Assay Kit (Beyotime Biotechnology, China, C1088) was used to detect the apoptosis in xenograft tumors according to the manufacture’s instruction. Fluorescence microscope BX53 (Olympus, Japan) was used to visualize the stained tissue sections.

### Statistical analysis

SPSS Version 16.0 (SPSS Inc., Chicago, IL) was used to analyze the results of experiments which were conducted in triplicate and presented as mean ± standard deviation (SD). The graphics were generated using GraphPad 6.0 (GraphPad Software, USA). Student’s *t*-test and one-way ANOVA were used to analyze the differences between two independent groups and multiple groups, respectively; Chi-square test was used to assess the differences of categorical data; *p* < 0.05 was considered statistically significant.

## Results

### Survivin and XIAP were overexpressed in colorectal cancer

**Fig. 1:**
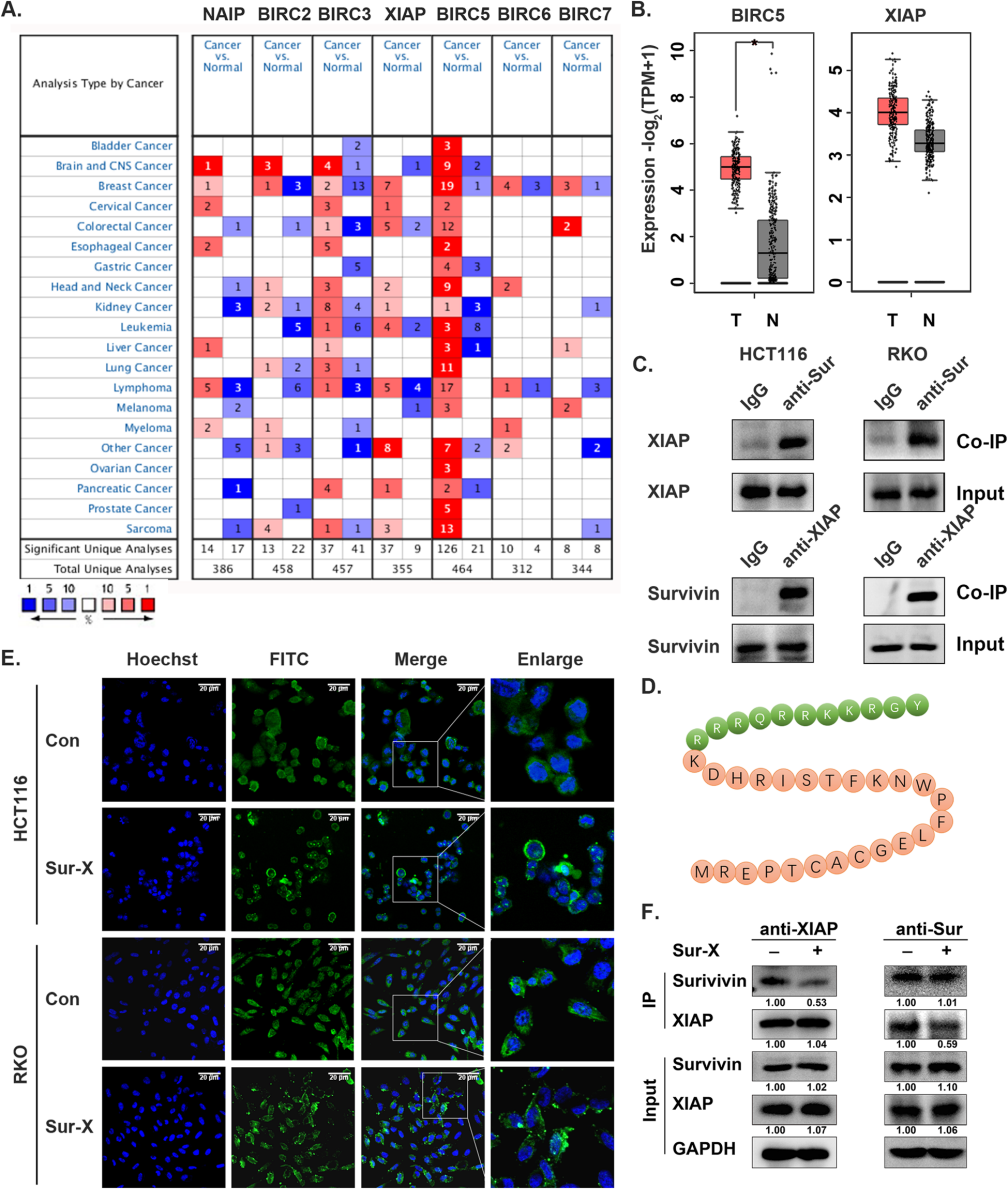
Peptide Sur-X targeted the survivin-XIAP complex in colorectal cancer cells. **a** The expression of IAPs in all cancer types available in Oncomine. **b** Analysis of survivin and XIAP expression in colorectal cancer by GEPIA (T = 275, *N* = 349). N, normal; T, tumor; *, *p* < 0.05. **c** The survivin-XIAP interaction in human colorectal cancer cells HCT116 and RKO, as determined by co-immunoprecipitation. Anti-Sur, anti-survivin antibody; anti-XIAP, anti-XIAP antibody. Three independent experiments were performed. **d** The amino acid sequence of Sur-X. Residues from TAT were labeled with green, those from survivin were labeled with orange. **e** Intracellular localization of FITC-Sur-X and FITC-Con. Scale bar, 20 μm. Three independent experiments were performed. **f** Effect of Sur-X on survivin-XIAP interaction as determined by co-immunoprecipitation. HCT116 cells were treated by 10 μM Sur-X for 1 h or not. Co-immunoprecipitation was performed by anti-XIAP antibody (left pannel) and anti-survivin antibody (right pannel), respectively. Survivin and XIAP were detected by Western blot analysis. GAPDH was used as a loading control. Anti-Sur, anti-survivin antibody; anti-XIAP, anti-XIAP antibody. Three independent experiments were performed

Online databases Oncomine and GEPIA were used to compare the expression of IAPs (NAIP, BIRC2, BIRC3, XIAP, BIRC5/survivin, BIRC6 and BIRC7, no data on BIRC8 was available) between colorectal cancer and normal tissues. As the most widely studied member of IAPs, survivin was significantly overexpressed in most cancers available in Oncomine including colorectal cancer, which was validated by the analysis of survivin expression profile in multiple cancers by GEPIA (Fig. 1a and Figure S1A). Furthermore, the analysis by GEPIA suggested that among other IAPs apart from survivin, the expression of XIAP was also higher in colorectal cancer tissues, although with no statistical significance (Fig. 1b and Figure S1B). These data indicated that survivin and XIAP are overexpressed in colorectal cancer and are potential therapeutic targets for colorectal cancer treatment.

### Peptide Sur-X was designed to target the survivin-XIAP complex

It has been reported that survivin and XIAP are able to form an IAP-IAP complex to stabilize both of them and synergistically antagonize the activity of Caspase 9 in cancer cells [23]. As shown in Fig. 1c, we performed co-immunoprecipitation of survivin and XIAP in colorectal cancer cells HCT116 and RKO, and found that survivin-immunocomplexes contained XIAP, and XIAP-immunocomplexes also contained survivin, demonstrating that these two proteins can form survivin-XIAP complex in colorectal cancer cells. Therefore, we inferred that blocking the interactions between survivin and XIAP might be a promising therapeutic strategy in colorectal cancer.

According to the finding of a previous study that the minimal XIAP-interacting region of survivin comprised residues K15-M38 [32], we designed and synthesized a peptide that targets the survivin-XIAP complex, Sur-X, which overlapped with residues of survivin from K15 to M38, and the TAT sequence was added to the N-terminal of the peptide to enhance its cell membrane permeability (Fig. 1d). Peptide Con consisted of the N-terminal TAT sequence and a random 24-amino acid sequence, used as a negative control.

To investigate the cell membrane permeability of the peptides, Sur-X and Con were labeled with FITC and the internalization of FITC-Sur-X and FITC-Con was observed within 30 min (Fig. 1e). Moreover, it was confirmed that Sur-X indeed disrupted the formation of survivin-XIAP complex in colorectal cancer cells. As shown in the left panel of Fig. 1f, less survivin was co-immunoprecipitated with XIAP in colorectal cancer cells treated by Sur-X for 1 h; similarly, the right panel indicated a significant reduction of XIAP in survivin-immunocomplexes in Sur-X-treated cells. These results suggested that Sur-X can interfere with the formation of survivin-XIAP complex in colorectal cancer cells and may have anticancer effects.

### Sur-X exhibited potent and specific anticancer activity in colorectal cancer cells

**Fig. 2:**
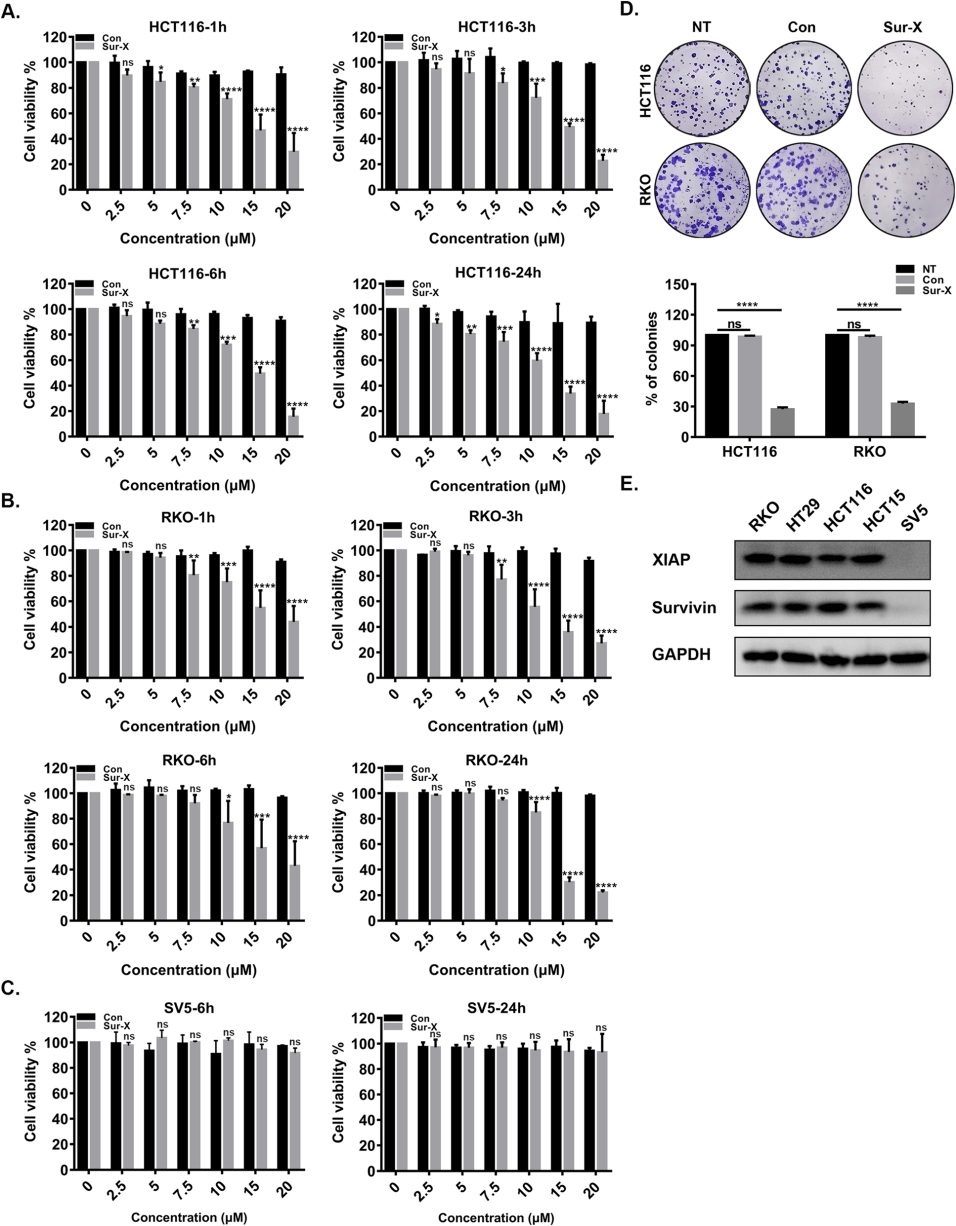
Sur-X exerted specific anticancer effect in colorectal cancer cells in vitro. **a**-**b** Cell viability was determined by MTT assay in human colorectal cancer cells HCT116 and RKO treated with indicated concentrations of Sur-X or Con for 1 h, 3 h, 6 h or 24 h. Mean and SD of three independent experiments are shown. **c** Cell viability was determined by MTT assay in human peritoneal mesothelial cell line SV5. Mean and SD of three independent experiments are shown. **d** Representative images of colonies formation in HCT116 and RKO cells with or without treatment of 10 μM Sur-X or Con (top). The number of colonies was presented as percentage of no-treatment group, mean and SD of three independent experiments are shown (bottom). NT, no treatment. **e** The expression of survivin and XIAP in RKO, HT29, HCT116, HCT15 and SV5 was detected by Western blot analysis. GAPDH was used as a loading control. Three independent experiments were performed. *, *p* < 0.05; **, *p* < 0.01; ***, *p* < 0.001; ****, *p* < 0.0001; ns, not significant

To evaluate the anticancer activity of Sur-X in colorectal cancer cells, four human colorectal cancer cell lines (HCT116, RKO, HCT15, and HT29) were exposed to a series of concentrations of Sur-X for 1, 3, 6, and 24 h, and their viabilities were assessed by MTT assay. Of note, viabilities of these four cell lines were significantly decreased in a dose-dependent manner with the treatment of Sur-X (Fig. 2a-b and Figure S2A-B). However, no inhibitory effect of Sur-X was observed in human peritoneal mesothelial cell line SV5 (Fig. 2c and Figure S2C). As the negative control, Con was ineffective in both colorectal cancer cell lines and normal cell line SV5 (Fig. 2a-c and Figure S2A-C). Similarly, the colony formation assay also confirmed the antitumor activity of Sur-X in colorectal cancer cells (Fig. 2d). Then, to explore whether the specific anticancer activity of Sur-X was dependent on survivin and XIAP, their expression levels in all the five cell lines were detected by Western blot analysis and it was found that both survivin and XIAP were highly expressed in colorectal cancer cells, but not detected in SV5 (Fig. 2e).

### Sur-X promoted colorectal cancer cell apoptosis by destabilizing survivin and XIAP

**Fig. 3:**
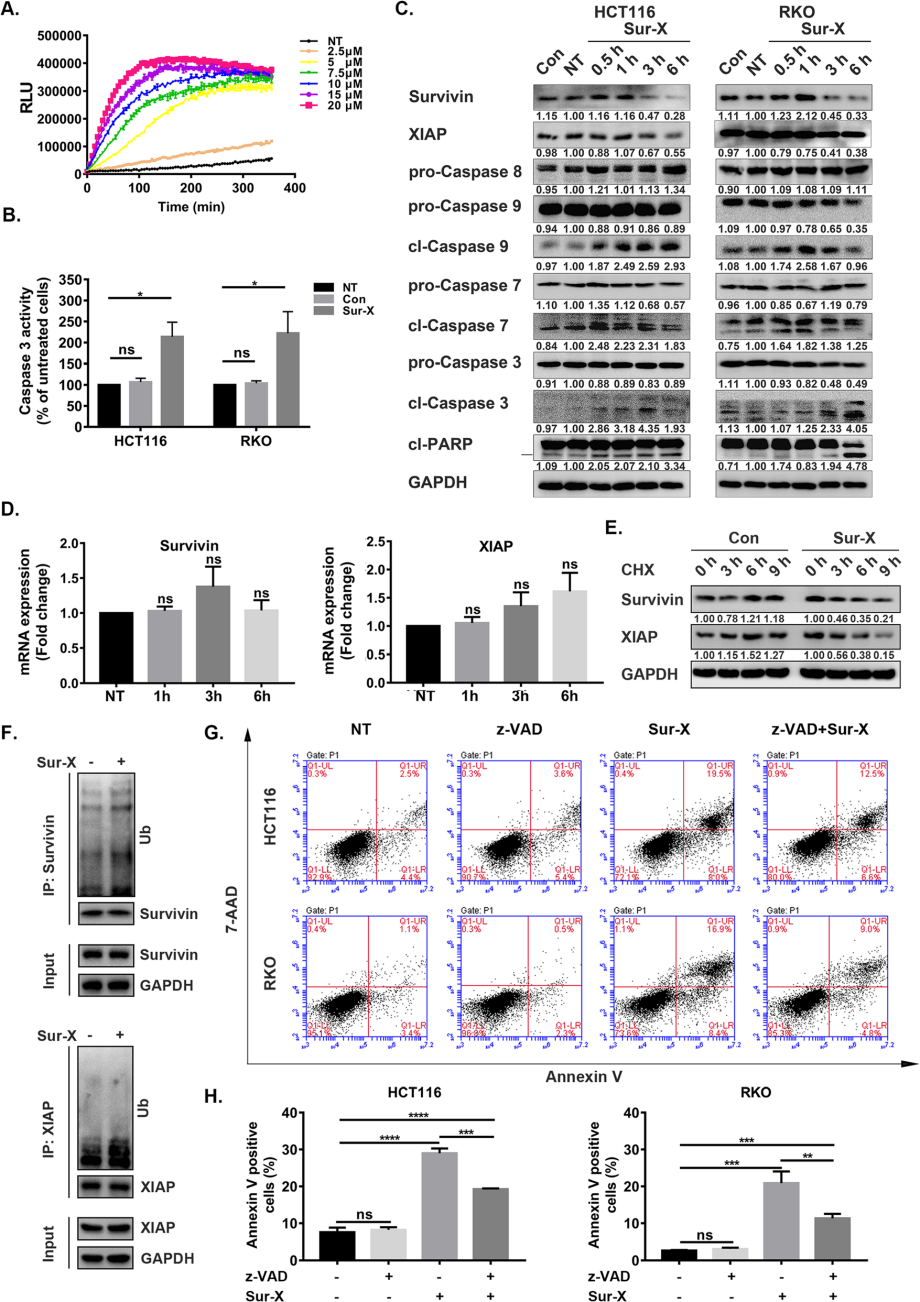
Sur-X induced apoptosis in colorectal cancer cells by promoting the degradation of survivin and XIAP. **a** The real-time detection of RLU (phosphatidylserine and Annexin V binding, apoptosis) in HCT116 cells over 6 h with indicated concentrations of Sur-X. NT, no treatment. Three independent experiments were performed. **b** The activity of Caspase 3 in HCT116 and RKO with or without treatment of 10 μM Sur-X or Con for 1 h. The relative Caspase 3 activity was presented as percentage of untreated group, mean and SD of three independent experiments are shown. NT, no treatment. **c** HCT116 and RKO cells were treated by 10 μM of Sur-X (0.5, 1, 3 and 6 h) or Con (6 h), the expression levels of apoptosis-related proteins were detected by Western blot analysis. GAPDH was used as a loading control. NT, no treatment. Three independent experiments were performed. **d** The effect of Sur-X on the mRNA expression of survivin and XIAP in HCT116 cells. The expression value was presented as fold change of untreated group, mean and SD of three independent experiments are shown. NT, no treatment. ns, not significant. **e** The protein expression of survivin and XIAP in HCT116 cells with the treatment of 10 μM Sur-X or Con in combination with 10 μg/ml CHX for indicated time. GAPDH was used as a loading control. Three independent experiments were performed. **f** The effect of Sur-X on the ubiquitination of survivin and XIAP. After pretreated by MG132 (10 μM) for 8 h, HCT116 cells were treated by 10 μM Sur-X for 1 h. Three independent experiments were performed. **g**-**h** Effect of z-VAD-pretreatment on Sur-X-induced cell death in HCT116 (top) and RKO (bottom) assessed by Annexin V/7-AAD assay (**g**). Quantification of Annexin V positive cells, mean and SD of three independent experiments are shown. NT, no treatment; z-VAD, cells were treated only by z-VAD; Sur-X, cells were treated by Sur-X (10 μM) for 6 h; z-VAD + Sur-X, cells were pretreated by z-VAD (50 μM) for 12 h and then treated by Sur-X in combination with z-VAD for another 6 h (**h**). *, *p* < 0.05; **, *p* < 0.01; ***, *p* < 0.001; ****, *p* < 0.0001; ns, not significant

Since the interactions between survivin and XIAP were decreased significantly upon the treatment of Sur-X for only 1 h (Fig. 1f), we speculated that Sur-X might induce apoptosis in colorectal cancer cells by destabilizing survivin and XIAP. Then, real-time apoptosis detection was performed to evaluate the apoptosis-inducing ability of Sur-X in HCT116 cells. As shown in Fig. 3a, a time-and dose-dependent significant increase in apoptotic signals (RLU) was observed in cells treated by Sur-X at indicated concentrations, except for 2.5 μM. Of note, Sur-X-induced apoptosis reached a plateau around 3 h at most concentrations (Fig. 3a), which was consistent with the finding that Sur-X rapidly inhibited the viability of colorectal cancer cells (Fig. 2a-b and Figure S2A-B). In addition, compared with untreated cells, significant increase in Caspase 3 activity was observed in Sur-X-treated cells (Fig. 3b). Further, the effects of Sur-X on the expression levels of apoptosis-related proteins were evaluated by Western blot. As shown in Fig. 3c, the cleavages of Caspase 9, 3, 7 and PARP were increased in both HCT116 and RKO cells treated with Sur-X, however, no activation of Caspase 8 was observed. Additionally, as expected, Sur-X decreased the expression of survivin and XIAP (Fig. 3c). Next, it was proved that Sur-X had no significant inhibitory effect on the mRNA expression of survivin and XIAP (Fig. 3d). However, when protein synthesis in colorectal cancer cells was blocked by CHX, Sur-X was found to promote the degradation of survivin and XIAP (Fig. 3e). Moreover, the ubiquitination levels of both survivin and XIAP were enhanced in colorectal cancer cells with the treatment of Sur-X (Fig. 3f). Additionally, it was noteworthy that Sur-X did not interfere with XIAP-Caspase 9 interactions (Figure S3). Together, these results indicated that the disruption of survivin-XIAP complex by Sur-X caused ubiquitination-mediated degradation of survivin and XIAP, and subsequently promoted Caspase 9-dependent intrinsic apoptosis in colorectal cancer cells.

Moreover, what is worth noting is that as shown in Fig. 3g and h, pre-treatment with z-VAD, a pan-caspase inhibitor, only partially rescued Sur-X-induced cell death in HCT116 and RKO cells, which indicated that apart from apoptosis, Sur-X might also induce other forms of programmed cell death.

### Sur-X induced necroptosis in colorectal cancer cells

**Fig. 4:**
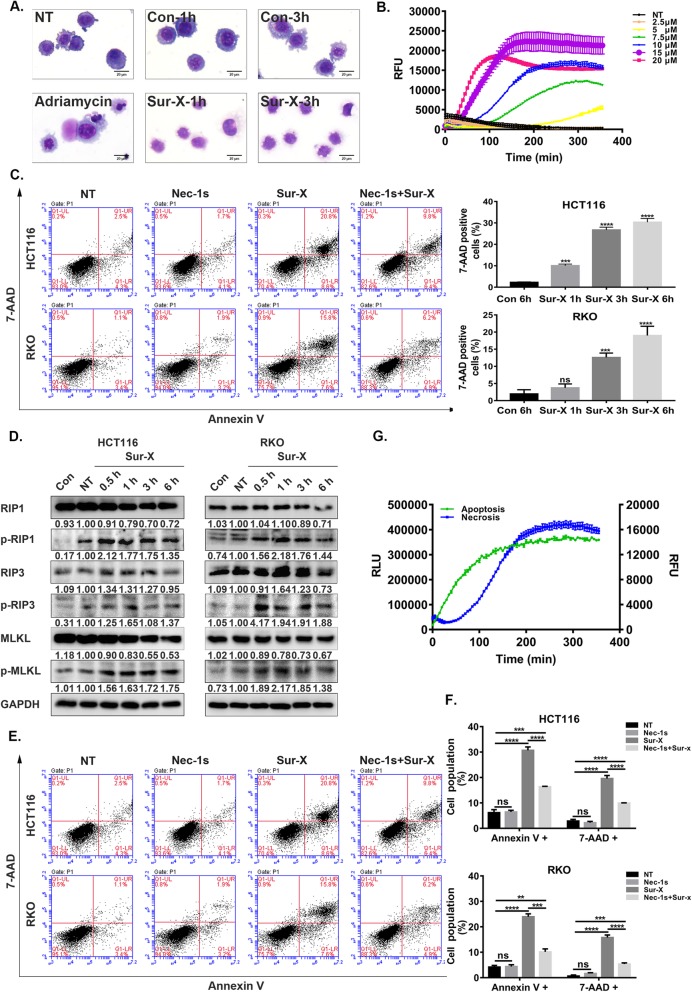
Sur-X promoted necroptosis in colorectal cancer cells. **a** Cell morphology as determined by Giemsa staining. After treated by 10 μM Sur-X or Con, HCT116 cells were stained with Giemsa and those treated by adriamycin (10 μM for 12 h) was used as a positive control of apoptosis. Scale bar, 50 μm. Three independent experiments were performed. **b** The real-time detection of RFU (cell membrane damage, necrosis) in HCT116 cells over 6 h with indicated concentrations of Sur-X. NT, no treatment. Three independent experiments were performed. **c** HCT116 (top) and RKO (bottom) cells were treated by 10 μM Sur-X for 1, 3 and 6 h, or Con for 6 h, and analyzed by Annexin V/7-AAD assay (left panel). Quantification of 7-AAD positive cells, mean and SD of three independent experiments are shown (right panel). **d** HCT116 and RKO cells were treated by 10 μM Sur-X (0.5, 1, 3 and 6 h) or Con (6 h), the expressions of necroptosis-related proteins were detected by Western blot analysis. GAPDH was used as a loading control. NT, no treatment. Three independent experiments were performed. **e-f** Effect of Nec-1 s-pretreatment on Sur-X-induced necroptosis in HCT116 (top) and RKO (bottom) assessed by Annexin V/7-AAD assay (**e**). Quantification of Annexin V positive cells and quantification of 7-AAD positive cells in HCT116 (top) and RKO (bottom), mean and SD of three independent experiments are shown. NT, no treatment; Nec-1 s, cells were treated only by Nec-1 s; Sur-X, cells were treated by Sur-X (10 μM) for 6 h; Nec-1 s + Sur-X, cells were pretreated by Nec-1 s (50 μM) for 12 h and treated by Sur-X in combination with Nec-1 s for another 6 h (**f**). **g** Kinetic detection of apoptosis (RLU, phosphatidylserine and Annexin V binding) and necroptosis (RFU, membrane integrity) in HCT116 cells treated by 10 μM Sur-X was conducted simultaneously over 6 h. Three independent experiments were performed. **, *p* < 0.01; ***, *p* < 0.001; ****, *p* < 0.0001; ns, not significant

To study whether Sur-X induced necroptosis in colorectal cancer cells, we first evaluated the effects of Sur-X on cell morphology by Giemsa staining. Of note, different from the classic apoptotic morphology (condensed chromatin and intact cell membrane) induced by adriamycin, necrotic morphology was observed in Sur-X-treated cells (Fig. 4a). In addition, real-time necrosis detection showed significant increase in RFU in cells treated by Sur-X in a dose- and time-dependent manner, and flow cytometry analysis presented a rapid increase of 7-AAD-positive cells in Sur-X-treated cells, which also suggested that Sur-X might induce necroptosis (Fig. 4b and c). Furthermore, expression levels of necroptosis-related proteins including Receptor-Interacting Protein 1 (RIP1), RIP3, Mixed Lineage Kinase Domain-Like (MLKL) and their phosphorylation in colorectal cancer cells following treatment with Sur-X were examined by Western blot analysis. As shown in Fig. 4d, after treatment with Sur-X for 30 min, p-RIP1, p-RIP3, and p-MLKL were all significantly increased. More importantly, Nec-1 s, a necroptosis inhibitor, partially attenuated Sur-X-induced cell death mainly by significantly reducing 7-AAD-positive cells, which also confirmed the contribution of necroptosis to the anticancer activity of Sur-X (Fig. 4e and f). In addition, rapid increases in necrotic signal (RFU) and apoptotic signal (RLU) were detected almost simultaneously in Sur-X-treated cells by real-time apoptosis and necrosis analysis (Fig. 4g and Figure S4A), which further distinguished necroptosis from secondary necrosis, the latter is manifested by a significant increase in RFU after the RLU has reached a plateau. Taken together, these data suggested that necroptosis also contributed to Sur-X-induced cell death.

### Sur-X disrupted XIAP-TAB1 interaction and accelerated TAK1 degradation

Since survivin could bind to the BIR1 and BIR3 domains of XIAP [23], we hypothesized that Sur-X might promote necroptosis by binding to XIAP to block the interactions between XIAP and other necroptosis-related essential proteins. Therefore, MD simulations were used to characterize the binding mode of Sur-X with XIAP. As shown in Table 1, 11 residues from Sur-X and 10 residues from XIAP formed 17 interaction pairs (the interactions between polar amino acids are mainly hydrogen bonds and those between non-polar amino acids are mainly hydrophobic interactions). Among the 10 residues from XIAP contributing to its binding with Sur-X, eight (L30, A34, S38, S45, D71, K85, N89, and F92) were located in the BIR1 domain (E26-I93). Thus, we speculated that Sur-X might interfere with the binding of other proteins to the BIR1 domain of XIAP.

**Table 1:** The predicted interacting residue pairs between Sur-X and XIAP

**Sur-X**	**XIAP**
R18	E266
T21	**S38**
F22	**A34**
N24	**S45**
W25	**L30, F92**
F27	**F92**
C31	**N89**
A32	**K85, N89**
C33	**N89**, T269
R37	**D71**
M38	**L30, S45, F92,** E266

Residues with bold font are in the BIR1 (E26-I93) of XIAP

**Fig. 5:**
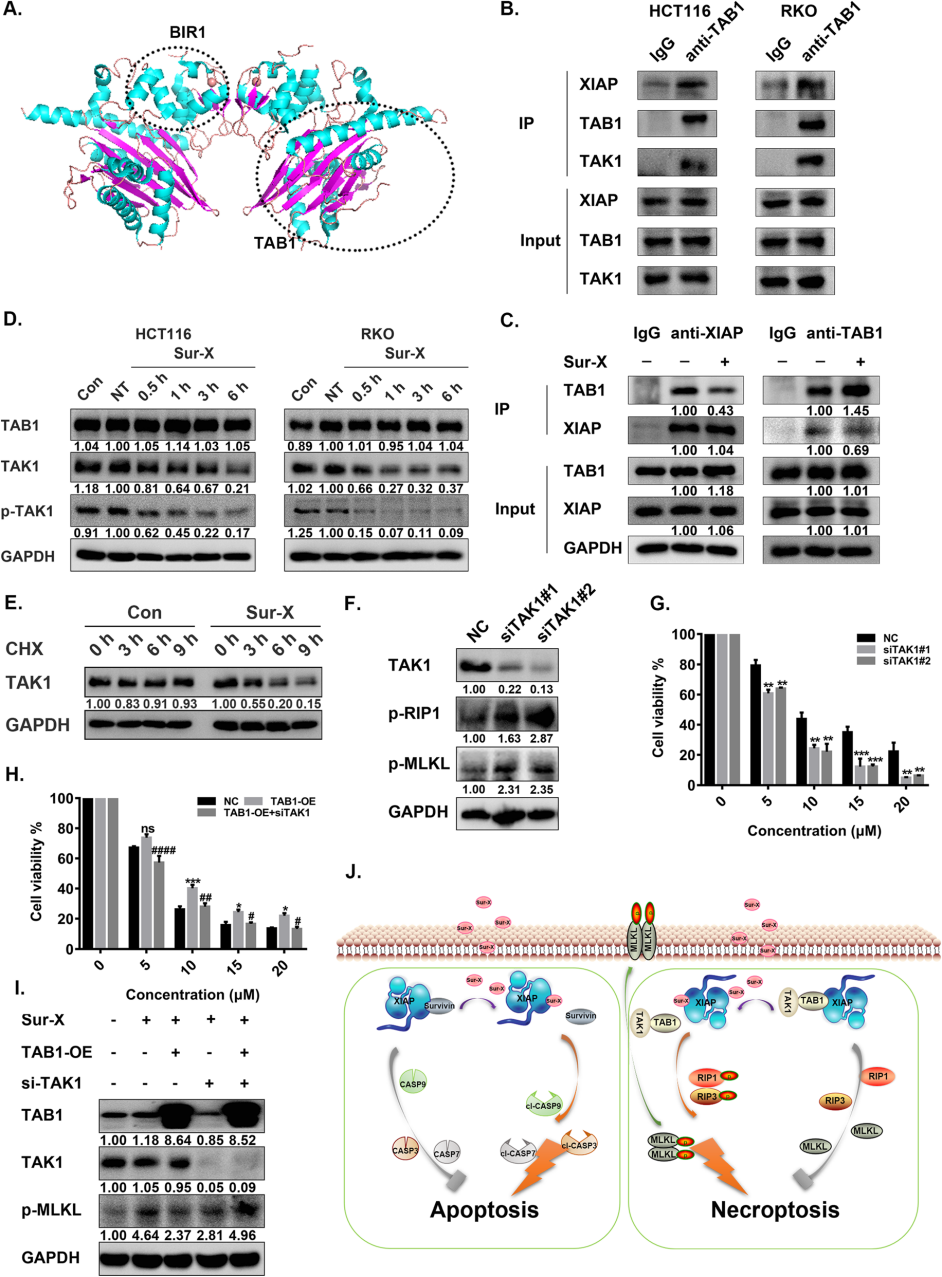
Sur-X induced necroptosis by blocking the interaction of XIAP and TAB1 in colorectal cancer cells. **a** The crystal structure of TAB1-BIR1 complex obtained from PDB (code: 2POP) and colored by PyMol according to secondary structure. Helix in cyan, sheet in magenta and loop in wheat. **b** Analysis of interactions between XIAP and TAB1, TAB1 and TAK1, in HCT116 and RKO cells by co-immunoprecipitation. Three independent experiments were performed. **c** Effect of Sur-X on XIAP-TAB1 interaction as determined by co-immunoprecipitation. HCT116 cells were treated by 10 μM Sur-X for 1 h or not. Co-immunoprecipitation was performed by anti-XIAP antibody and anti-TAB1 antibody, respectively. XIAP and TAB1 were detected by Western blot analysis. GAPDH was used as a loading control. Anti-XIAP, anti-XIAP antibody; anti-TAB1, anti-TAB1 antibody. Three independent experiments were performed. **d** HCT116 and RKO cells were treated by 10 μM Sur-X (0.5, 1, 3 and 6 h) or Con (6 h), the expressions of TAB1, TAK1 and p-TAK1 were detected by Western blot analysis. GAPDH was used as a loading control. NT, no treatment. Three independent experiments were performed. **e** The protein expression of TAK1 in HCT116 cells with the treatment of 10 μM Sur-X or Con in combination with 10 μg/ml CHX for indicated time. GAPDH was used as a loading control. Three independent experiments were performed. **f**-**g** HCT116 was transiently transfected with NC and TAK1 siRNAs for 48 h, the expression of TAK1, p-RIP1 and p-MLKL were detected by Western blot analysis. GAPDH was used as a loading control. Three independent experiments were performed (**f**). HCT116 was transiently transfected with NC and TAK1 siRNAs for 48 h, followed by treatment of Sur-X for 6 h, and cell viability was detected by MTT assay, mean and SD of three independent experiments are shown. **, *p* < 0.01; ***, *p* < 0.001 (**g**). **h-i** Effect of TAB1 and TAK1 expression on the anticancer activity of Sur-X in HCT116 cells through necroptosis. HCT116 was transiently transfected with pcDNA3.1(+) and TAB1-OE plasmids for 24 h, followed by transfection of NC and TAK1 siRNA for 48 h. Transfected cells were treated by Sur-X for another 6 h and cell viability was detected by MTT assay, mean and SD of three independent experiments are shown. Comparison with NC: *, *p* < 0.05; ***, *p* < 0.001; ns, not significant. Comparison with TAB1-OE: #, *p* < 0.05; ##, *p* < 0.01; ####, *p* < 0.0001 (**h**). The expressions of TAB1, TAK1 and p-MLKL were detected by Western blot analysis. GAPDH was used as a loading control. TAB1-OE, TAB1-overexpression. Three independent experiments were performed (**i**). **j** Schematic representation of the anticancer mechanism of Sur-X

Interestingly, by searching for “XIAP BIR1” in the PDB database, it was found that the BIR1 of XIAP could interact with TAB1 (Fig. 5a). Besides, a previous study reported that XIAP-TAB1 interactions were crucial for the activation of TAK1, an endogenous inhibitor of necroptosis [33, 34]. Thus, it was plausible that Sur-X might disrupt the XIAP-TAB1 interactions to relieve the inhibition of TAK1 on necroptosis. To this end, we first confirmed the formation of the XIAP-TAB1-TAK1 complex in colorectal cancer cells by the detection of XIAP and TAK1 in co-immunoprecipitation with TAB1 (Fig. 5b). Then, it was demonstrated that the interactions between XIAP and TAB1 were significantly decreased in cells with the treatment of Sur-X for 1 h (Fig. 5c). Taken together, this set of experiments suggested that Sur-X can block the interactions between XIAP and TAB1. Additionally, since it was found that the protein levels of both TAK1 and p-TAK1 were significantly decreased in Sur-X treated cells, whereas no change in TAB1 was observed (Fig. 5d), we speculated that disruption of XIAP-TAB1 interaction by Sur-X might promote the degradation of TAK1. As expected, compared with Con, Sur-X significantly accelerated the degradation of TAK1 (Fig. 5e).

### Overexpression of TAB1 attenuated Sur-X-induced necroptosis, but knockdown of TAK1 sensitized colorectal cancer cells to Sur-X

To further identify the role of TAB1 and TAK1 in Sur-X-induced necroptosis, expressions of TAK1 and TAB1 in colorectal cancer cells were altered alone or in combination. Of note, knockdown of TAK1 increased the levels of p-RIP1 and p-MLKL and enhanced the anticancer activity of Sur-X in colorectal cancer cells (Fig. 5f-g and Figure S4B-C). However, TAB1 overexpression (TAB1-OE) attenuated the inhibition effects of Sur-X on colorectal cancer cells and decreased the expression of p-MLKL, which further confirmed the disruption of XIAP-TAB1 interactions by Sur-X (Fig. 5h-i and Figure S4D-E). Moreover, knockdown of TAK1 restored the anticancer effect of Sur-X and p-MLKL expression in TAB1-OE cells (Fig. 5h-i and Figure S4D-E). All these results indicated that Sur-X promoted necroptosis by interfering with the interactions between XIAP and TAB1 and subsequently destabilizing TAK1 to alleviate the inhibition of TAK1 on necroptosis in colorectal cancer cells.

In addition, considering the contribution of XIAP-TAB1-TAK1 complex to NF-κB activation [33], the effects of Sur-X on NF-κB signaling pathway were detected. As shown in Figure S5A, after treated by Sur-X for 30 min or1 h, the expression levels of p-IκB and p-P65 in colorectal cancer cells showed a transient increase. Interestingly, the most significant increase of TNF-α secreted by colorectal cancer cells was observed after the treatment of Sur-X for 30 min (Figure S5B). Thus, it was plausible that Sur-X induced a rapid and transient secretion of TNF-α by colorectal cancer cells and subsequently activated NF-κB signaling pathway for a short time.

### Sur-X induced both apoptosis and necroptosis in vivo

**Fig. 6:**
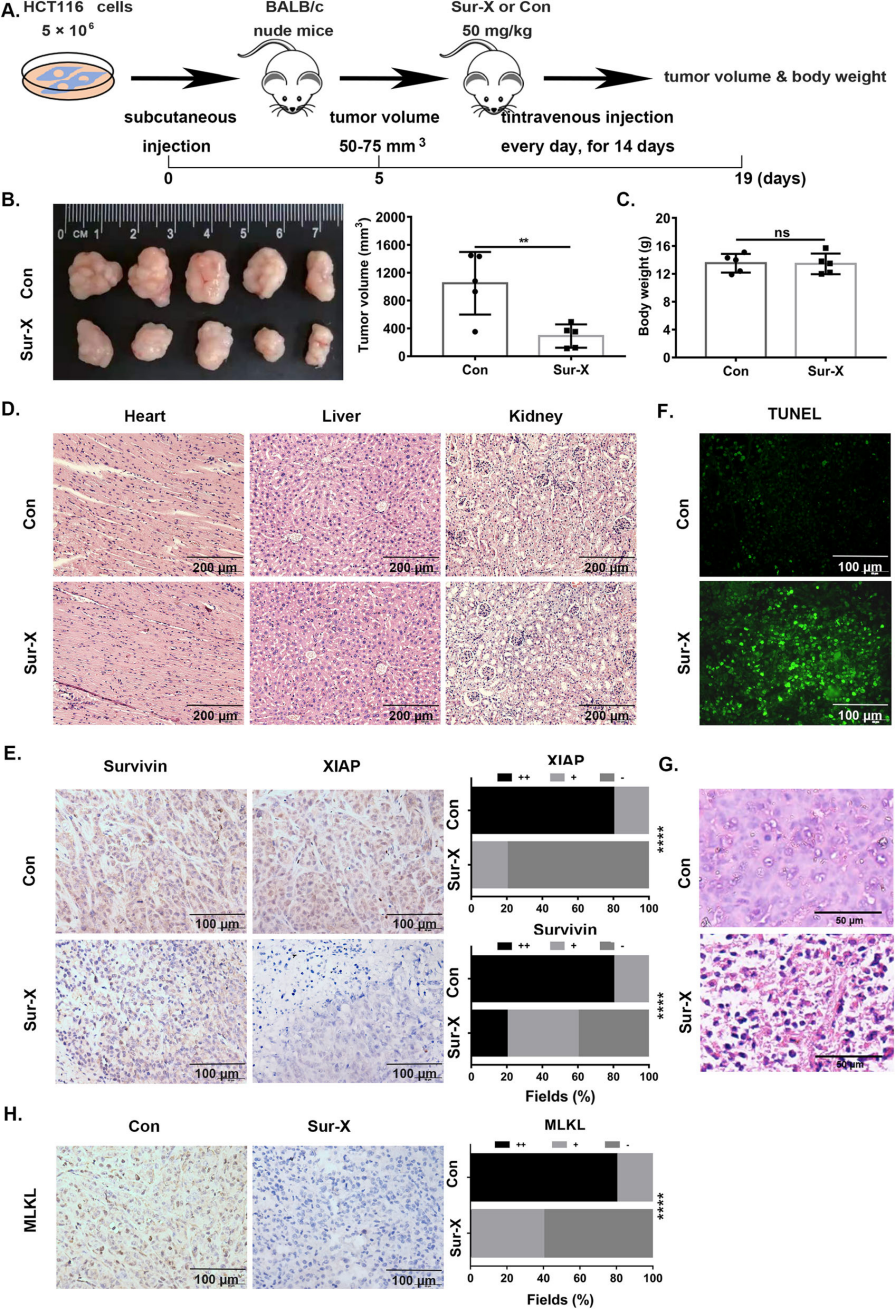
Sur-X inhibited tumor growth in xenograft mouse models. **a** Schematic of subcutaneous xenograft mouse model with intravenous injection. **b** Picture (left) and volume (right) of tumor nodules of mice from Sur-X group and Con group. *n* = 5; **, *p* < 0.01. **c** Body weight of mice from two groups. *n* = 5; ns, not significant. **d** Representative HE staining sections of heart, liver and kidney of mice from Sur-X group and Con group. Three independent experiments were performed. Scale bars, 200 μm. **e** Representative IHC staining of survivin, XIAP in tumors of mice from two groups (left), and 5 non-overlapping fields were randomly selected for the analysis of their expression in two groups, Chi-square test was performed to examine the difference (right). Scale bars, 100 μm. ****, *p* < 0.0001. Three independent experiments were performed. **f** TUNEL staining in tumors of mice from two groups. Scale bars, 100 μm. Three independent experiments were performed. **g** The representative HE staining pictures of tumor tissues from two groups. Scale bar, 50 μm. Three independent experiments were performed. **h** Representative IHC staining of MLKL in tumors of mice from two groups, and 5 non-overlapping fields were randomly selected for the analysis of MLKL expression in two groups, Chi-square test was performed to examine the difference (right). Scale bars, 100 μm. ****, *p* < 0.0001. Three independent experiments were performed

To validate the anticancer efficacy and safety of Sur-X in vivo, subcutaneous xenograft mouse model was established and received intravenous injection of Sur-X and Con (Fig. 6a). Of note, significant inhibition on the growth of tumors was observed in mice with the treatment of Sur-X (Fig. 6b). Importantly, no significant difference in body weight was found between Sur-X-treated mice and Con group, and no toxicity of Sur-X to vital organs such as heart, liver and kidneys was observed (Fig. 6c and d). Furthermore, the decreased expression of survivin and XIAP and enhanced staining of TUNEL were observed in tumor tissues from mice treated by Sur-X, which indicated the induction of apoptosis by Sur-X in vivo (Fig. 6e, f and Figure S6A). In addition, the in vivo pro-necroptosis effect of Sur-X was confirmed by the extensive necrosis and the reduced MLKL expression in tumor tissues from Sur-X-treated mice (Figure S6B, Fig. 6g and h).

Altogether, in vitro and in vivo data indicated that Sur-X exerted specific and potent anticancer effects by promoting apoptosis and necroptosis in colorectal cancer cells.

## Discussion

Apoptosis evasion is one of the most important hallmarks of tumorigenesis and a major contributor of chemotherapy resistance in various cancers including colorectal cancer [35]. Thus, inducing apoptosis has been considered as one of the fundamental strategies in cancer treatment [36]. Survivin is believed a promising target in cancer therapy due to its apoptosis inhibition function and specific overexpression in most cancers [37]. To date, plenty of survivin-targeted anticancer drugs have been developed to interfere with the interactions between survivin and other proteins [18]. In particular, anticancer peptides have emerged with potential for use in cancer treatment, due to their ease of synthesis, less toxic side-effects and lower immunogenicity [38]. Shepherdin is a potent anticancer peptidomimetic antagonist of the survivin-Hsp90 complex, which can occupy the ATP-pocket of Hsp90 [16]. It was reported that shepherdin exerted significant pro-apoptotic effects mainly by promoting rapid degradation of survivin in multiple cancer cell lines and xenograft mouse models [16, 39, 40]. However, since Hsp90 also chaperones some tumor suppressive proteins such as interferon regulatory factor 1, LATS1 and LATS2 kinases, shepherdin’s inhibition on Hsp90 might deregulate tumor suppressor pathways, which might be one of the reasons why shepherdin failed in clinical application [41]. Therefore, it is necessary to develop more specific and effective peptides targeting survivin with clinical translation potential.

In this study, we focused on the interaction between survivin and XIAP. It was reported that survivin and XIAP can form a heterocomplex to stabilize both of them and synergistically antagonize the activity of Caspase 9 to inhibit apoptosis in cancer cells [23]. Therefore, we speculated that the peptide targeting the survivin-XIAP complex might be a promising therapeutic strategy for colorectal cancer treatment. As the first peptide that targets the survivin-XIAP complex, Sur-X was proved to be able to interfere with survivin-XIAP interaction, promote ubiquitination-mediated degradation of survivin and XIAP, and hence induce Caspase 9-dependent intrinsic apoptosis in colorectal cancer cells. Of note, previous study has reported that XIAP can directly inhibit Caspase 9 through the BIR 3 domain [42], however, MD simulations in our study showed that Sur-X mainly bound to the BIR1 of XIAP. Moreover, no reduction of XIAP-Caspase 9 interaction was detected in Sur-X-treated cells. Thus, it is plausible that Sur-X induces intrinsic apoptosis via disrupting the survivin-XIAP complex and subsequently accelerating the degradation of survivin and XIAP, rather than directly interfering with XIAP-Caspase 9 interaction.

As an alternative of caspase-dependent apoptosis, necroptosis is a novel form of regulated cell death with a necrotic morphology, mainly mediated by RIP1, RIP3 and MLKL [43]. Our study found that apart from inducing apoptosis, Sur-X also promoted necroptosis both in colorectal cancer cells and HCT116 xenograft mouse models. In fact, shepherdin was also reported to be able to induce non-apoptotic cell death, whereas the exact form was not identified [16]. Similar to our findings, LCL161, a SMAC mimetic compound which can bind and antagonize IAPs including XIAP, was observed to promote both apoptosis and necroptosis in breast cancer cells [44]. BV6, another small-molecule SMAC mimetic, was able to trigger necroptosis in pancreatic cancer cells when caspase activation was blocked [45]. Yabal et al. reported that loss of XIAP resulted in increased expression and aberrantly elevated ubiquitination of RIP1 to promote RIP3-dependent cell death [46]. However, whether XIAP directly interacts with RIP1 and/or RIP3 remains unknown. Therefore, the exact mechanisms by which XIAP regulates necroptosis in cancer cells merit further investigation.

In the present study, no elevated expression of RIP1 was observed in Sur-X-treated cells, which excluded the assumption that Sur-X might regulate RIP1 by reducing XIAP expression. Of note, TAK1, a member of the serine/threonine protein kinase family, has recently been known as an endogenous necroptosis inhibitor [47]. It was reported that TAK1 can directly mediate inhibitory phosphorylation of RIP1 on S321 to prevent RIP1-RIP3 interaction [48]. The TAK1-dependent S25 phosphorylation of RIP1 by IKKα/β was also demonstrated to inhibit the kinase activity of RIP1 [49]. Podder B and his colleagues reported that inhibiting TAK1 can promote necroptosis through RIP1 activation and necrosome formation in melanoma cells [34]. Moreover, it was found that TAB1, an upstream adaptor for the activation of the kinase TAK1, can interact with the BIR1 domain of XIAP [33]. In our study, the result of MD simulations indicated that Sur-X mainly bound to the BIR1 domain of XIAP. Thus, we hypothesized that Sur-X might interfere with the interaction between the BIR1 domain of XIAP and TAB1. As expected, Sur-X was found to be able to effectively inhibit the interaction between XIAP and TAB1 in the XIAP-TAB1-TAK1 complex. Interestingly, the disruption of XIAP-TAB1 interaction by Sur-X accelerated the degradation of TAK1 and hence the inhibition of TAK1 on necroptosis was attenuated in colorectal cancer cells. Taken together, apart from inducing intrinsic apoptosis by disrupting the survivin-XIAP complex, the novel anticancer peptide Sur-X can also promote necroptosis by accelerating the degradation of TAK1 via inhibiting XIAP-TAB1 interaction.

## Conclusions

In summary, we designed a novel anticancer peptide, Sur-X, which targets the survivin-XIAP complex, and found that Sur-X can promote both apoptosis and necroptosis via interfering with the interaction between survivin and XIAP, XIAP and TAB1, respectively. Since survivin, XIAP, TAB1 and TAK1 are involved in the anticancer effects of Sur-X, it can be inferred that Sur-X may have broader efficacy in cancer patients with high expression of survivin, XIAP, TAB1 and/or TAK1. Moreover, Sur-X may also disrupt the interactions between other essential proteins with survivin or XIAP, which need further exploration. Similar to most other peptide agents, the poor stability of Sur-X in vivo may limit its translational application for cancer patients. Therefore, in the future study, we will utilize available manufacturing technologies to improve the bioavailability of Sur-X. In addition, conjugation with other anticancer agents is considered as another effective approach to facilitate the clinical translation of Sur-X.

## Supplementary information

**Additional file 1: Table S1.** Primers used in qRT-PCR assay.

**Additional file 2: Figure S1.** Analysis of IAPs expression by the online database GEPIA.

**Additional file 3: Figure S2.** Specific anticancer effect of Sur-X in colorectal cancer cells.

**Additional file 4: Figure S3.** Effect of Sur-X on XIAP-Caspase 9 interaction.

**Additional file 5: Figure S4.** Sur-X promoted necroptosis in RKO.

**Additional file 6: Figure S5.** The effect of Sur-X on NF-κB signaling pathway.

**Additional file 7: Figure S6.** Sur-X promoted both apoptosis and necroptosis in vivo.

## Data Availability

All data generated or analyzed during this study are included in this published article.

## Abbreviations

IAP: Inhibitor of apoptosis protein
BIR: Baculovirus IAP repeat
CPC: Chromosomal passenger complex
GEPIA: Gene Expression Profiling Interactive Analysis
z-VAD: z-VAD-fmk
SV5: Human peritoneal mesothelial cell line HMrSV5
RLU: Relative luminescence units
RFU: Relative fluorescence units
aMD: Accelerated molecular dynamics
MD: Molecular dynamics
CHX: Cycloheximide
HE: Haematoxylin and eosin
TCGA: The Cancer Genome Atlas
TAB1-OE: TAB1 overexpression
