# Correction to: Sur-X, a novel peptide, kills colorectal cancer cells by targeting survivin-XIAP complex

**DOI:** 10.1186/s13046-021-02182-4

**Published:** 2021-12-07

**Authors:** Wanxia Fang, Xiaofang Che, Guohui Li, Anhui Wang, Yizhe Wang, Xiaonan Shi, Kezuo Hou, Xiaojie Zhang, Xiujuan Qu, Yunpeng Liu

**Affiliations:** 1 Department of Medical Oncology, The First Hospital of China Medical University, Shenyang, 110001 China; 2 Key Laboratory of Anticancer Drugs and Biotherapy of Liaoning Province, The First Hospital of China Medical University, Shenyang, 110001 China; 3 Liaoning Province Clinical Research Center for Cancer, The First Hospital of China Medical University, Shenyang, 110001 China; 4 Key Laboratory of Precision Diagnosis and Treatment of Gastrointestinal Tumors, Ministry of Education, The First Hospital of China Medical University, Shenyang, 110001 China; 5 Laboratory of Molecular Modeling and Design, State Key Laboratory of Molecular Reaction Dynamics, Dalian Institute of Chemical Physics, Chinese Academy of Sciences, Dalian, 116024 China; 6 State Key Laboratory of Fine Chemicals, School of Chemistry, Dalian University of Technology, Dalian, 116024 China; 7 Department of Respiratory and Infectious Disease of Geriatrics, The First Hospital of China Medical University, Shenyang, 110001 China

**Correction to: J Exp Clin Cancer Res 39, 82 (2020)**

**https://doi.org/10.1186/s13046-020-01581-3**

Following publication of the original article [1], the authors identified some minor errors in Fig. 4, specifically:

- In Fig. 4c, left panel, the original figure presented a misused result of the analysis of Annexin V/7-AAD assay.

**Fig. 4:**
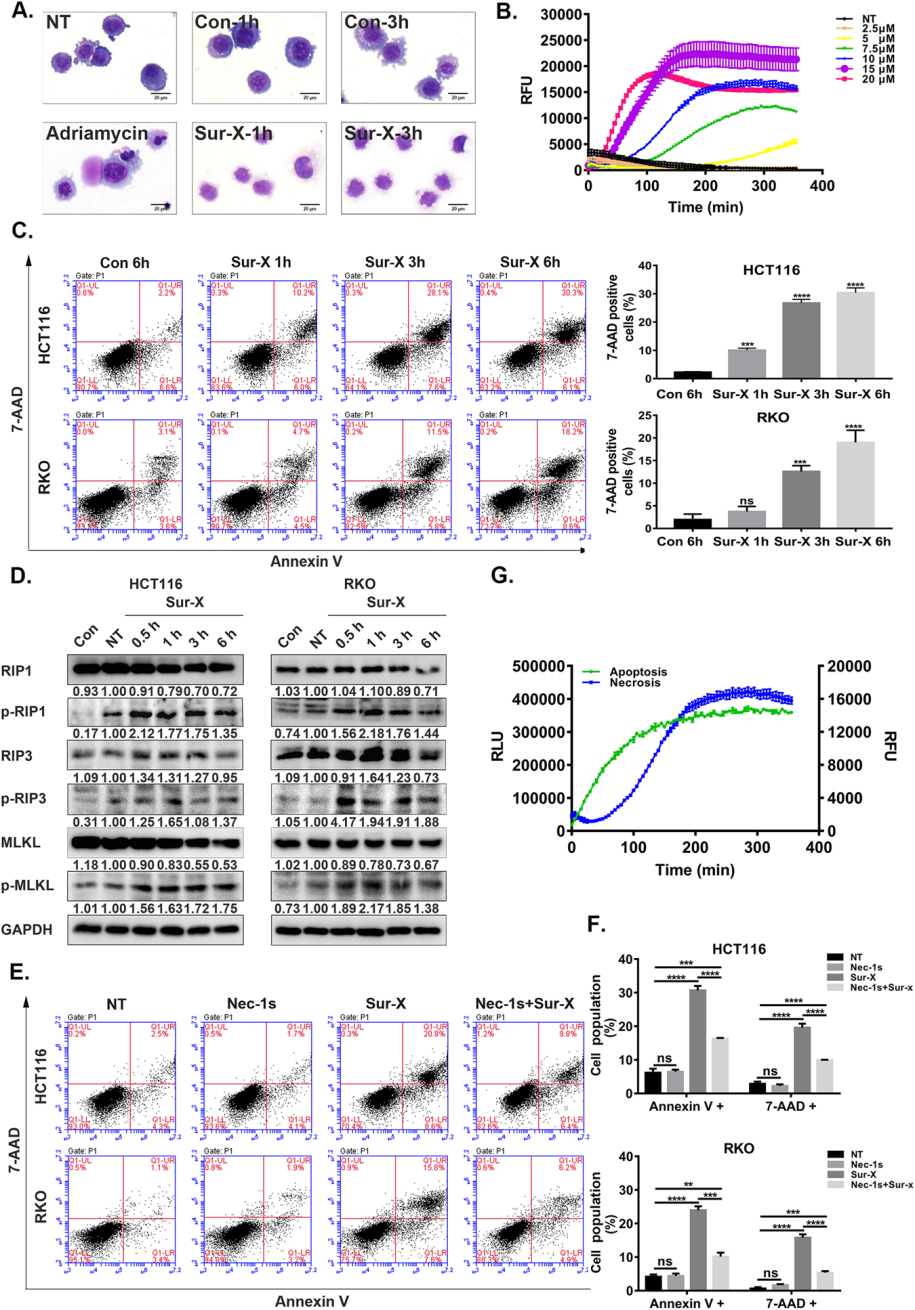
Sur-X promoted necroptosis in colorectal cancer cells. **a** Cell morphology as determined by Giemsa staining. After treated by 10 μM Sur-X or Con, HCT116 cells were stained with Giemsa and those treated by adriamycin (10 μM for 12 h) was used as a positive control of apoptosis. Scale bar, 50 μm. Three independent experiments were performed. **b** The real-time detection of RFU (cell membrane damage, necrosis) in HCT116 cells over 6 h with indicated concentrations of Sur-X. NT, no treatment. Three independent experiments were performed. **c** HCT116 (top) and RKO (bottom) cells were treated by 10 μM Sur-X for 1, 3 and 6 h, or Con for 6 h, and analyzed by Annexin V/7-AAD assay (left panel). Quantification of 7-AAD positive cells, mean and SD of three independent experiments are shown (right panel). **d** HCT116 and RKO cells were treated by 10 μM Sur-X (0.5, 1, 3 and 6 h) or Con (6 h), the expressions of necroptosis-related proteins were detected by Western blot analysis. GAPDH was used as a loading control. NT, no treatment. Three independent experiments were performed. **e-f** Effect of Nec-1 s-pretreatment on Sur-X-induced necroptosis in HCT116 (top) and RKO (bottom) assessed by Annexin V/7-AAD assay (**e**). Quantification of Annexin V positive cells and quantification of 7-AAD positive cells in HCT116 (top) and RKO (bottom), mean and SD of three independent experiments are shown. NT, no treatment; Nec-1 s, cells were treated only by Nec-1 s; Sur-X, cells were treated by Sur-X (10 μM) for 6 h; Nec-1 s + Sur-X, cells were pretreated by Nec-1 s (50 μM) for 12 h and treated by Sur-X in combination with Nec-1 s for another 6 h (**f**). **g** Kinetic detection of apoptosis (RLU, phosphatidylserine and Annexin V binding) and necroptosis (RFU, membrane integrity) in HCT116 cells treated by 10 μM Sur-X was conducted simultaneously over 6 h. Three independent experiments were performed. **, *p* < 0.01; ***, *p* < 0.001; ****, *p* < 0.0001; ns, not significant

The corrected figure is given here. The corrections do not have any effect on the final conclusions of the paper. The original article has been corrected.
