# Supplementary material for: Sur-X, a novel peptide, kills colorectal cancer cells by targeting survivin-XIAP complex
Source: J Exp Clin Cancer Res. 2020 May 7;39:82. doi: 10.1186/s13046-020-01581-3 (PMC7203900; doi:10.1186/s13046-020-01581-3)
Supplement: Supplementary file 1 — Additional file 1: Table S1. Primers used in qRT-PCR assay. [file 13046_2020_1581_MOESM1_ESM.docx]

**Table S1. Primers used in qRT-PCR assay**

| Target | Forward (5’-3’) | Reverse (5’-3’) |
| --- | --- | --- |
| Survivin | AGGACCACCGCATCTCTACAT | AAGTCTGGCTCGTTCTCAGTG |
| XIAP | TATCAGACACCATATACCCGAGG | TGGGGTTAGGTGAGCATAGTC |
