# Supplementary material for: Sur-X, a novel peptide, kills colorectal cancer cells by targeting survivin-XIAP complex
Source: J Exp Clin Cancer Res. 2020 May 7;39:82. doi: 10.1186/s13046-020-01581-3 (PMC7203900; doi:10.1186/s13046-020-01581-3)

**Supplementary Figure S1.** **Analysis of IAPs expression by the online database GEPIA**

(A) The expression profile of survivin in all cancer types available in GEPIA. Red front, significant high expression; green front, significant low expression; black front, no differential expression. (B) The expression of other members of IAPs in colorectal cancer. *, *p* < 0.05.
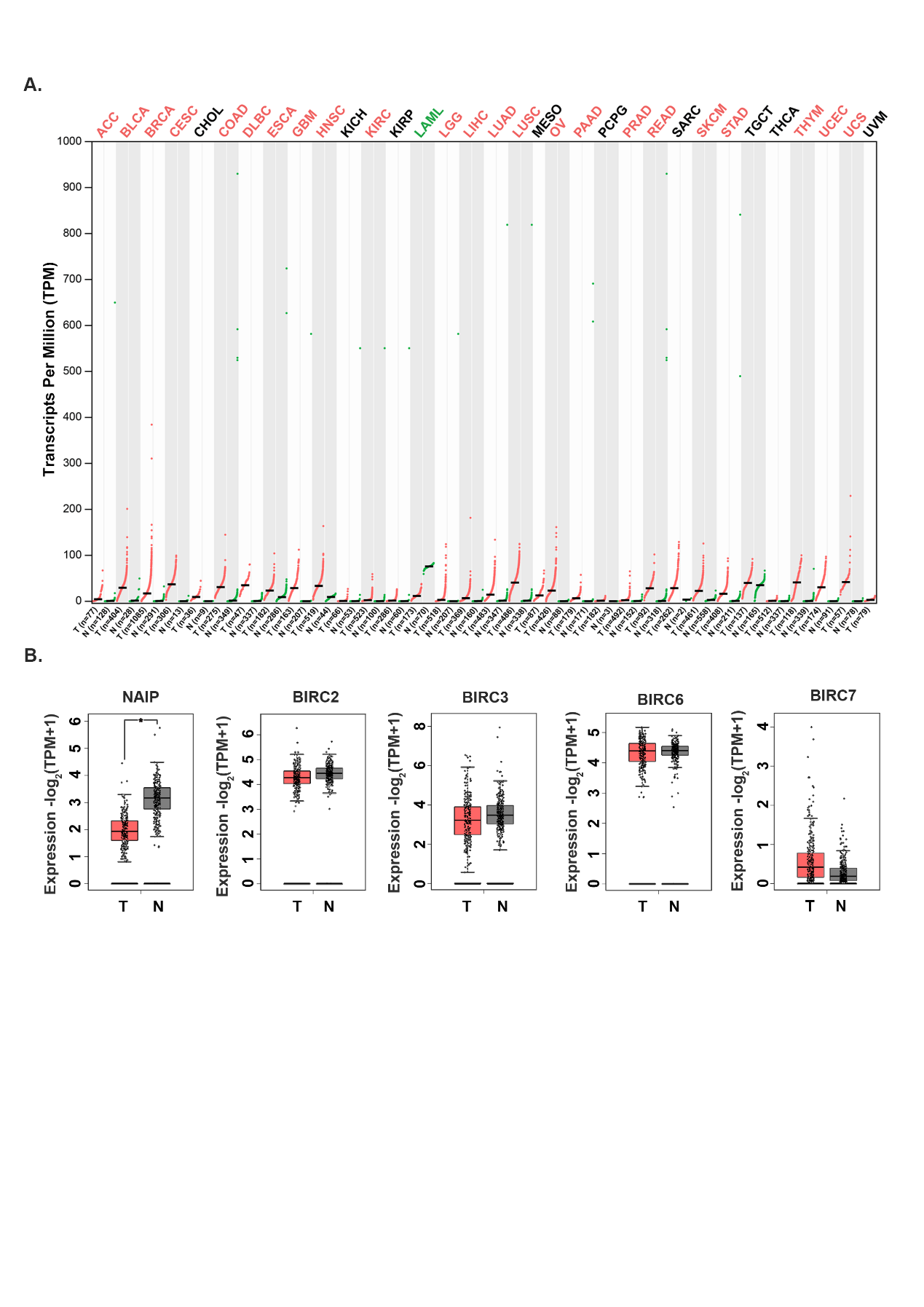

Supplement: Supplementary file 2 — Additional file 2: Figure S1. Analysis of IAPs expression by the online database GEPIA. [file 13046_2020_1581_MOESM2_ESM.docx]
