# Supplementary material for: Sur-X, a novel peptide, kills colorectal cancer cells by targeting survivin-XIAP complex
Source: J Exp Clin Cancer Res. 2020 May 7;39:82. doi: 10.1186/s13046-020-01581-3 (PMC7203900; doi:10.1186/s13046-020-01581-3)

**Supplementary Figure S2.** **Specific anticancer effect of Sur-X in colorectal cancer cells**

(A-B) Cell viability was determined by MTT assay in human colorectal cancer cells HCT15 and HT29 treated with indicated concentrations of Sur-X or Con for 1 h, 3 h, 6 h or 24 h. Mean and SD of three independent experiments are shown. (C) Cell viability was determined by MTT assay in human peritoneal mesothelial cell line SV5. Mean and SD of three independent experiments are shown. **p* < 0.05, ***p* < 0.01, ****p* < 0.001, *****p* < 0.0001, ns, not significant.


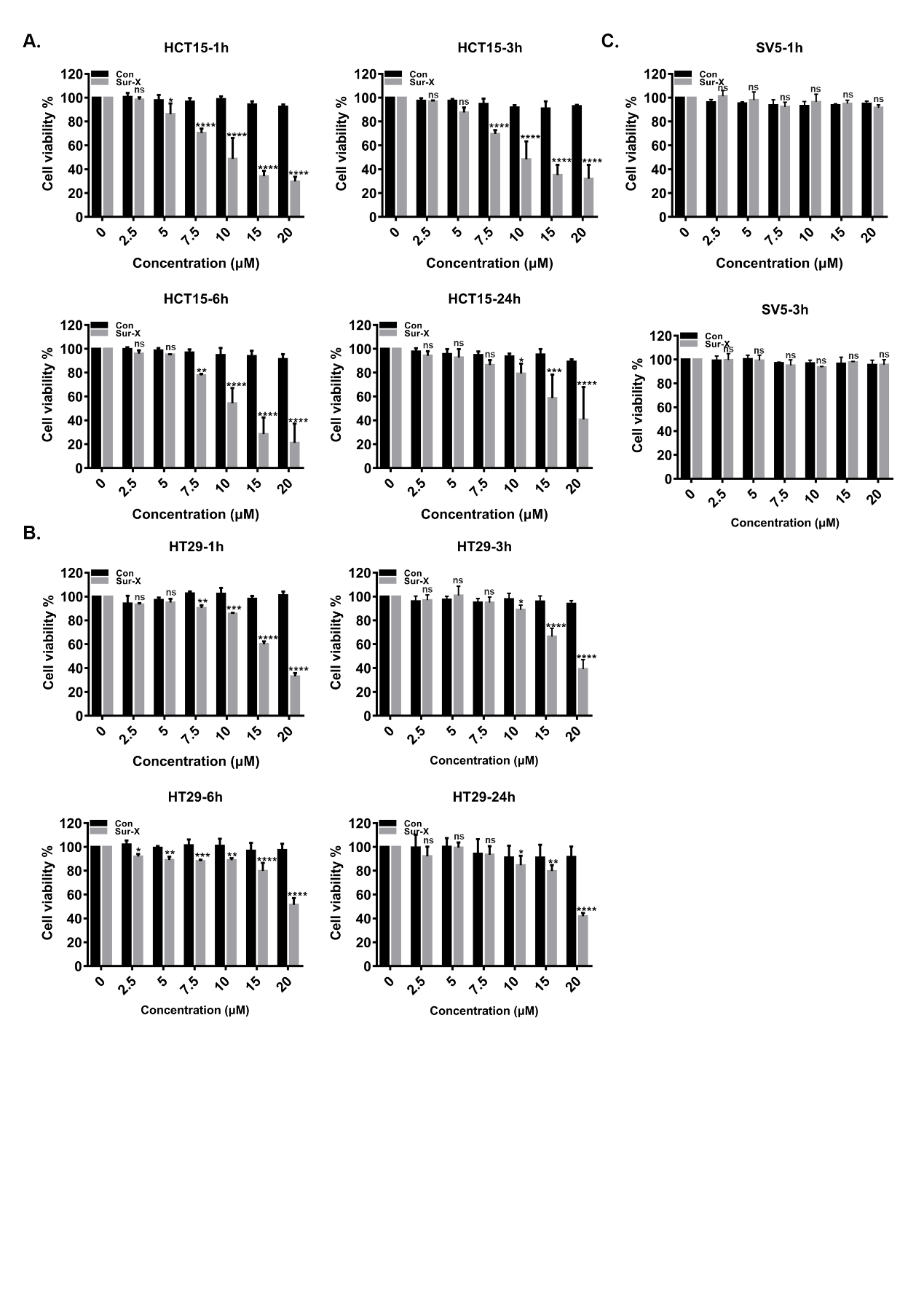

Supplement: Supplementary file 3 — Additional file 3: Figure S2. Specific anticancer effect of Sur-X in colorectal cancer cells. [file 13046_2020_1581_MOESM3_ESM.docx]
