# Supplementary material for: Sur-X, a novel peptide, kills colorectal cancer cells by targeting survivin-XIAP complex
Source: J Exp Clin Cancer Res. 2020 May 7;39:82. doi: 10.1186/s13046-020-01581-3 (PMC7203900; doi:10.1186/s13046-020-01581-3)

**Supplementary Figure S3.** **Effect of Sur-X on XIAP-Caspase 9 interaction**

HCT116 cells were treated by 10 μM Sur-X for 1 h or not. Co-immunoprecipitation was performed by anti-XIAP antibody, XIAP and Caspase 9 were detected by Western blot analysis. GAPDH was used as a loading control. Three independent experiments were performed.


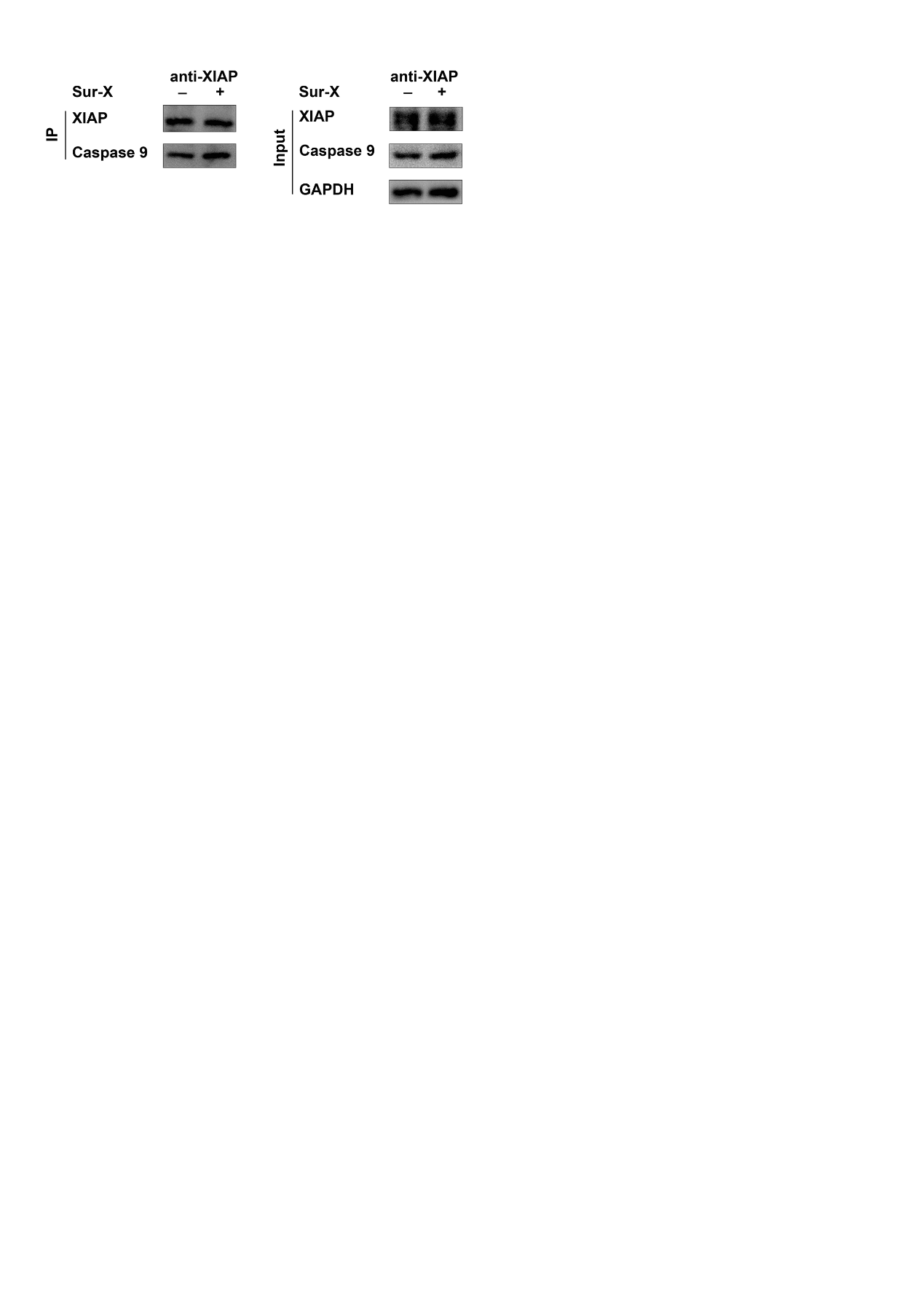

Supplement: Supplementary file 4 — Additional file 4: Figure S3. Effect of Sur-X on XIAP-Caspase 9 interaction. [file 13046_2020_1581_MOESM4_ESM.docx]
