# Supplementary material for: Sur-X, a novel peptide, kills colorectal cancer cells by targeting survivin-XIAP complex
Source: J Exp Clin Cancer Res. 2020 May 7;39:82. doi: 10.1186/s13046-020-01581-3 (PMC7203900; doi:10.1186/s13046-020-01581-3)

**Supplementary Figure S4.** **Sur-X promoted necroptosis in RKO**

(A) Kinetic detection of apoptosis (RLU, phosphatidylserine and Annexin V binding) and necroptosis (RFU, membrane integrity) in RKO cells treated by 10 μM Sur-X was conducted simultaneously over 6 h. Three independent experiments were performed. (B) RKO was transiently transfected with NC and TAK1 siRNAs for 48 h, the expression of TAK1, p-RIP1 and p-MLKL were detected by Western blot analysis. GAPDH was used as a loading control. Three independent experiments were performed. (C) RKO was transiently transfected with NC and TAK1 siRNAs for 48 h, followed by treatment of Sur-X for 6 h, and cell viability was detected by MTT assay, mean and SD of three independent experiments are shown. *, *p* < 0.05; **, *p* < 0.01; ns, not significant. (D)-(E) Effect of TAB1 and TAK1 expression on the anticancer activity of Sur-X in RKO cells through necroptosis. RKO was transiently transfected with pcDNA3.1(+) and TAB1-OE plasmids for 24 h, followed by transfection of NC and TAK1 siRNA for 48 h. Transfected cells were treated by Sur-X for another 6 h and cell viability was detected by MTT assay, mean and SD of three independent experiments are shown. Comparison with NC: **, *p* < 0.01; ***, *p* < 0.001; ns, not significant. Comparison with TAB1-OE: ^#^, *p* < 0.05; ^##^, *p* < 0.01 (D). The expression of TAB1, TAK1 and p-MLKL were detected by Western blot analysis. APDH was used as a loading control. TAB1-OE, TAB1-overexpression. Three independent experiments were performed (E).


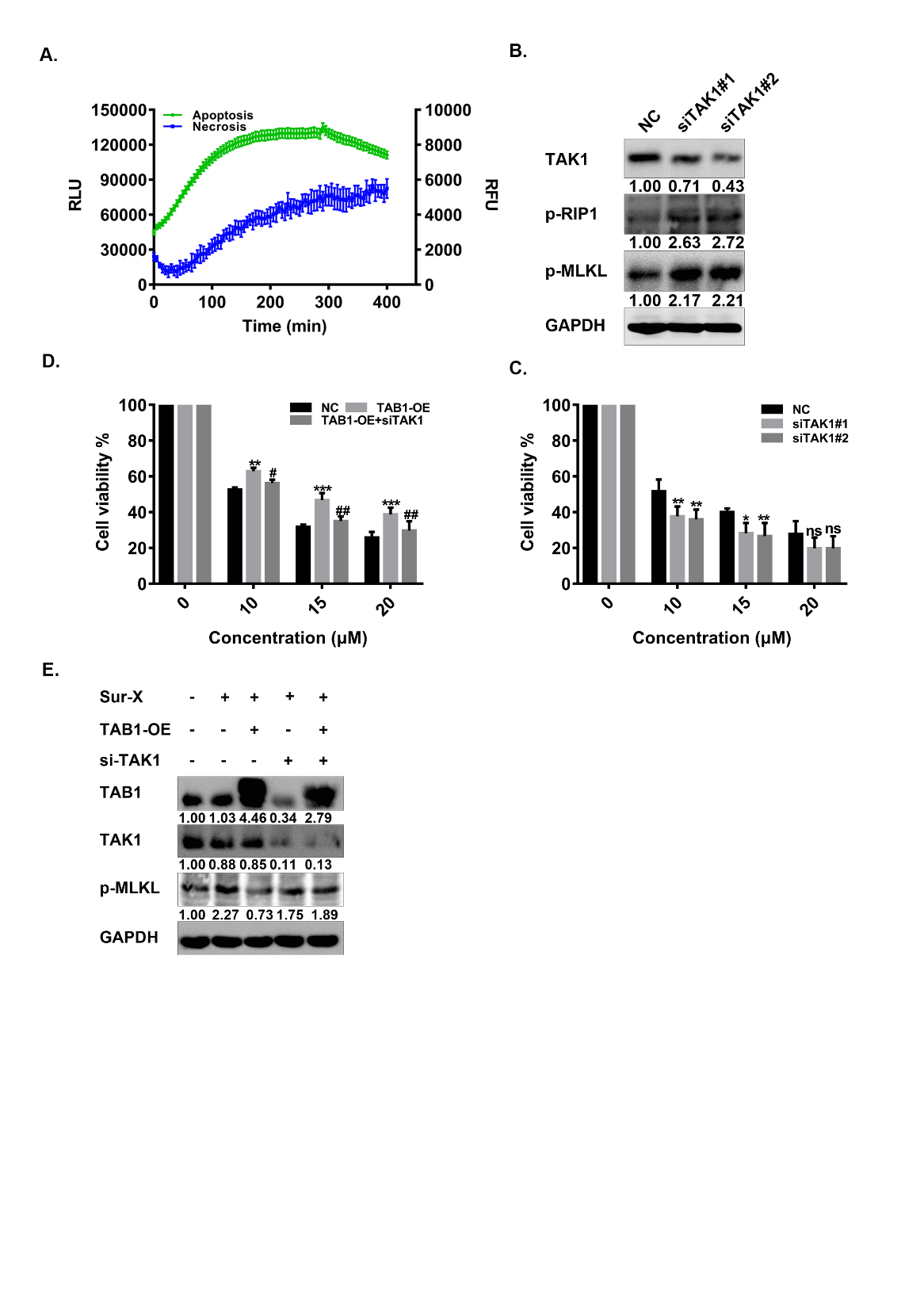

Supplement: Supplementary file 5 — Additional file 5: Figure S4. Sur-X promoted necroptosis in RKO. [file 13046_2020_1581_MOESM5_ESM.docx]
