# Supplementary material for: Sur-X, a novel peptide, kills colorectal cancer cells by targeting survivin-XIAP complex
Source: J Exp Clin Cancer Res. 2020 May 7;39:82. doi: 10.1186/s13046-020-01581-3 (PMC7203900; doi:10.1186/s13046-020-01581-3)

**Supplementary Figure S5.** **The effect of Sur-X on** **NF-κB signaling pathway**

(A) HCT116 and RKO cells were treated by 10 μM Sur-X (0.5, 1, 3 and 6 h) or Con (6 h), status of NF-κB signaling pathway was detected by Western blot analysis. GAPDH was used as a loading control. NT, no treatment. Three independent experiments were performed. (B) HCT116 cells were treated by 10 μM Sur-X (0.5, 1, 3 and 6 h) or Con (6 h), TNF-α in cell supernatants was detected by ELISA and the concentration was normalized by total protein (μg). Mean and SD of three independent experiments are shown. NT, no treatment; **, *p* < 0.01; ***, *p* < 0.001; ****, *p* < 0.0001; ns, not significant.


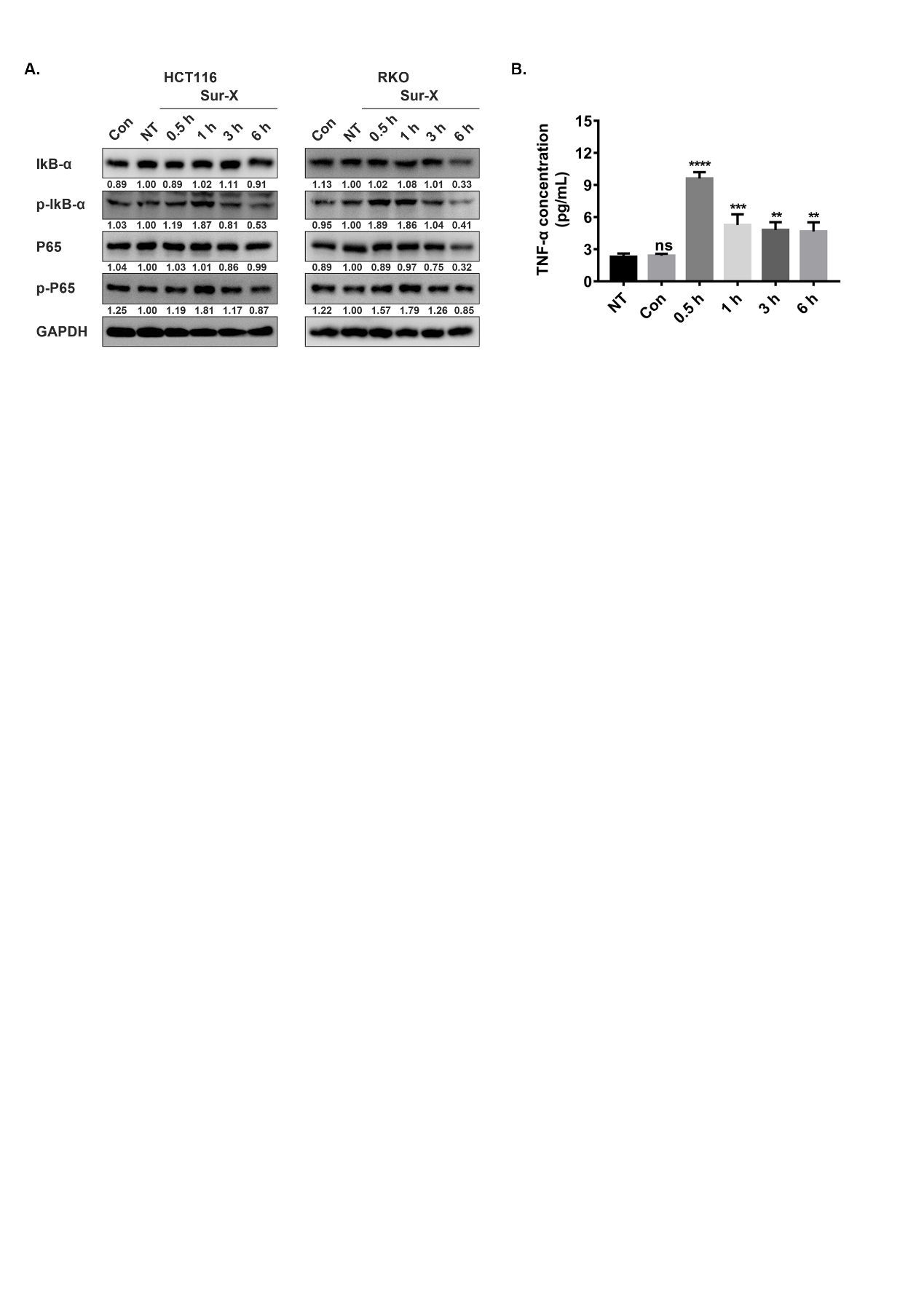

Supplement: Supplementary file 6 — Additional file 6: Figure S5. The effect of Sur-X on NF-κB signaling pathway. [file 13046_2020_1581_MOESM6_ESM.docx]
