# Supplementary material for: Sur-X, a novel peptide, kills colorectal cancer cells by targeting survivin-XIAP complex
Source: J Exp Clin Cancer Res. 2020 May 7;39:82. doi: 10.1186/s13046-020-01581-3 (PMC7203900; doi:10.1186/s13046-020-01581-3)

**Supplementary Figure S6.** **Sur-X promoted both apoptosis and necroptosis *in vivo***

(A) Quantitative analysis of TUNEL staining in tumors of mice from two groups (n=5). (B) Quantitative analysis of necrotic area in tumors of mice from two groups (n=5). **, *p* < 0.01; ****, *p* < 0.0001.


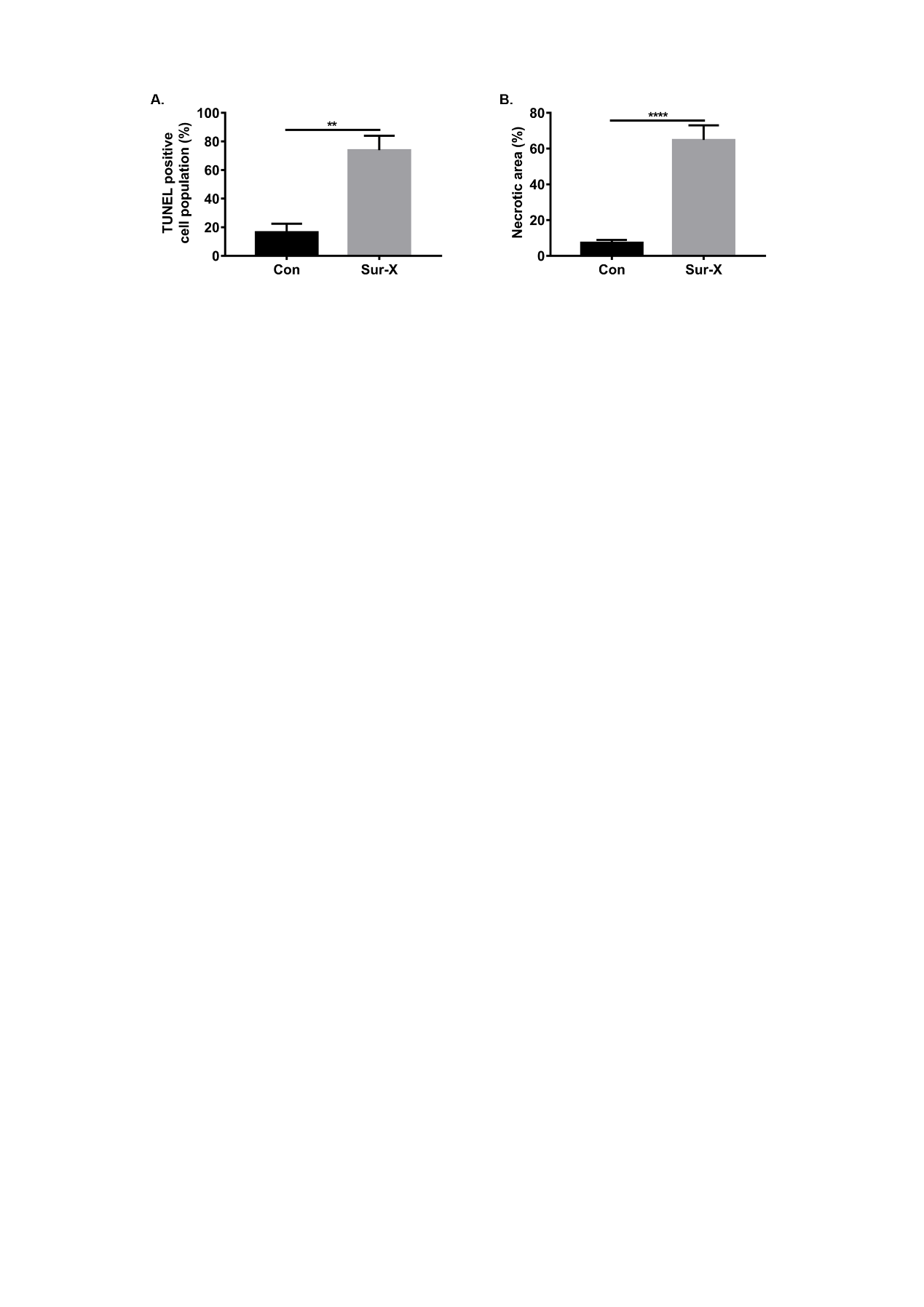

Supplement: Supplementary file 7 — Additional file 7: Figure S6. Sur-X promoted both apoptosis and necroptosis in vivo. [file 13046_2020_1581_MOESM7_ESM.docx]
